# BB0562 is a nutritional virulence determinant with lipase activity important for *Borrelia burgdorferi* infection and survival in fatty acid deficient environments

**DOI:** 10.1371/journal.ppat.1009869

**Published:** 2021-08-20

**Authors:** Hunter W. Kuhn, Amanda G. Lasseter, Philip P. Adams, Carlos Flores Avile, Brandee L. Stone, Darrin R. Akins, Travis J. Jewett, Mollie W. Jewett

**Affiliations:** 1 Division of Immunity and Pathogenesis, Burnett School of Biomedical Sciences, University of Central Florida College of Medicine, Orlando, Florida, United States of America; 2 Division of Molecular and Cellular Biology, Eunice Kennedy Shriver National Institute of Child Health and Human Development, Bethesda, Maryland, United States of America; 3 Postdoctoral Research Associate Program, National Institute of General Medical Sciences, National Institute of Health, Bethesda, Maryland, United States of America; 4 Department of Microbiology and Immunology, University of Oklahoma Health Sciences Center, Oklahoma City, Oklahoma, United States of America; University of Montana, UNITED STATES

## Abstract

The Lyme disease spirochete *Borrelia burgdorferi* relies on uptake of essential nutrients from its host environments for survival and infection. Therefore, nutrient acquisition mechanisms constitute key virulence properties of the pathogen, yet these mechanisms remain largely unknown. *In vivo* expression technology applied to *B*. *burgdorferi* (BbIVET) during mammalian infection identified gene *bb0562*, which encodes a hypothetical protein comprised of a conserved domain of unknown function, DUF3996. DUF3996 is also found across adjacent encoded hypothetical proteins BB0563 and BB0564, suggesting the possibility that the three proteins could be functionally related. Deletion of *bb0562*, *bb0563* and *bb0564* individually and together demonstrated that *bb0562* alone was important for optimal disseminated infection in immunocompetent and immunocompromised mice by needle inoculation and tick bite transmission. Moreover, *bb0562* promoted spirochete survival during the blood dissemination phase of infection. Gene *bb0562* was also found to be important for spirochete growth in low serum media and the growth defect of Δ*bb0562 B*. *burgdorferi* was rescued with the addition of various long chain fatty acids, particularly oleic acid. In mammals, fatty acids are primarily stored in fat droplets in the form of triglycerides. Strikingly, addition of glyceryl trioleate, the triglyceride form of oleic acid, to the low serum media did not rescue the growth defect of the mutant, suggesting *bb0562* may be important for the release of fatty acids from triglycerides. Therefore, we searched for and identified two canonical GXSXG lipase motifs within BB0562, despite the lack of homology to known bacterial lipases. Purified BB0562 demonstrated lipolytic activity dependent on the catalytic serine residues within the two motifs. In sum, we have established that *bb0562* is a novel nutritional virulence determinant, encoding a lipase that contributes to fatty acid scavenge for spirochete survival in environments deficient in free fatty acids including the mammalian host.

## Author summary

*Borrelia burgdorferi*, the causative agent of Lyme disease, has a small genome and lacks the ability to synthesize essential nutrients on its own as well as many of the virulence properties typical of bacterial pathogens that contribute to disease. The clinical manifestations of Lyme disease predominantly result from inflammation in response to the *B*. *burgdorferi* infection. Therefore, nutrient acquisition functions constitute key virulence factors for the pathogen. Fatty acids are critical components of *B*. *burgdorferi* membranes and lipoproteins, which the spirochete must scavenge from the host environment. Previously, through a genetic screen for *B*. *burgdorferi* genes that are expressed during mammalian infection we identified gene of unknown function, *bb0562*. Herein, we demonstrate that *bb0562* encodes a lipase that plays a role in the release of free fatty acids from triglycerides. Furthermore, *bb0562* contributes to *B*. *burgdorferi* survival and dissemination in the mammalian host. BB0562 is important for spirochete survival in environments low in free fatty acids thereby adding to *B*. *burgdorferi*’s arsenal of nutritional virulence determinants necessary for the pathogen to be maintained in the tick-mouse enzootic cycle and to cause disseminated disease.

## Introduction

Lyme disease, caused by the spirochete *Borrelia* (*Borreliella*) *burgdorferi*, is the leading vector-borne bacterial illness in the world [1]. *B*. *burgdorferi* is naturally maintained in an enzootic cycle between an arthropod vector, *Ixodes scapularis* ticks, and diverse small vertebrate reservoir hosts [2]. Humans become infected with *B*. *burgdorferi* through the bite of an infected tick [3]. If left untreated, *B*. *burgdorferi* disseminates from the bite site, transiently through the blood, to distal tissues leading to the debilitating clinical manifestations of Lyme disease such as arthritis, carditis, facial nerve palsy and encephalopathy [4].

For *B*. *burgdorferi* to achieve a disseminated infection the pathogen must adapt to physical conditions of the host environment, scavenge critical nutrients, overcome host innate and adaptive immune defenses, and interact with key host cell molecules to effectively move through and colonize distal tissues [5,6]. Broad research efforts in the field have resulted in identification of numerous *B*. *burgdorferi* proteins important for spirochete dissemination [5,7]. Largely, these studies have been guided by hypotheses based on protein sequence identity and conserved domain analyses. However, *B*. *burgdorferi* harbors a reduced genome lacking many canonical metabolic, virulence, and host defense evasion proteins. Furthermore, a large number of *B*. *burgdorferi* genes encode hypothetical proteins of unknown function which are uniquely conserved among *Borrelia* species [8,9]. Together these observations suggest that *B*. *burgdorferi* has evolved novel mechanisms to survive in the host.

As a result of its condensed genome, *B*. *burgdorferi* has significantly reduced metabolic and biosynthetic capabilities [9,10] and therefore is an obligate pathogen, dependent on its tick vector and mammalian host for salvage of essential nutrients. The competition between pathogens and the metabolism of their hosts for limited nutrients in the host environment has been coined “nutritional virulence” [11]. There is a growing body of evidence that nutrient acquisition functions are key virulence determinants for *B*. *burgdorferi* [12]. Uptake systems for glucose [13], oligopeptides [14], purines [15], manganese [16] and riboflavin [17] are essential for mammalian infection. Manganese uptake is also required for *B*. *burgdorferi* survival in ticks [16]. In addition, glycerol utilization is critical for spirochete persistence in ticks [18]. *B*. *burgdorferi* lacks genes for the de novo biosynthesis of fatty acids and cholesterol [9,10], which are critical components of the spirochete membrane, lipoproteins, glycolipids and lipid rafts [19–24]. *B*. *burgdorferi* has been shown to acquire cholesterol from eukaryotic cells through direct contact [21], yet the genetic basis for this process remains unknown. Gene *bb0646* encodes a lipase with specificity for saturated and polyunsaturated substrates, as well as has hemolytic activity [25], suggesting a possible role in fatty acid acquisition. The lipolytic activity of BB0646 contributes to optimal infectivity of *B*. *burgdorferi* [25], supporting fatty acid scavenge as a virulence property of the spirochete.

In order to elucidate novel factors important for *B*. *burgdorferi* survival and infection we previously sought to identify transcripts expressed by the pathogen during an active mammalian infection through application of *in vivo* expression technology (BbIVET) [26,27]. This *in vivo* genetic screen identified 233 infection-active putative promoter sequences, referred to as *B*. *b**urgdorferi* *i**n* *v**ivo* expressed sequences (*Bbives*). Ninety-one *Bbives* mapped within 300 nts upstream of annotated start codons for open reading frames (ORFs) [27,28], suggesting that these sequences serve as promoters for the proximal genes during infection. Approximately 33% of the 91 *Bbives* mapped upstream of genes annotated to encode hypothetical proteins of unknown function. Many of these genes have yet to be investigated for their contributions to *B*. *burgdorferi* infection.

Herein, we targeted the BbIVET-identified gene of unknown function, *bb0562* and putatively related adjacent genes *bb0563* and *bb0564* for deletion and examination throughout the *B*. *burgdorferi* enzootic cycle. We demonstrated that of these three genes, *bb0562* alone was important for disseminated infection in mice. We established that *bb0562* is a novel nutritional virulence determinant, encoding a lipase that contributes to fatty acid scavenge for spirochete survival in fatty acid deficient environments including the mammalian host.

## Results

### BbIVET-identified gene *bb0562* is constitutively expressed throughout the *B*. *burgdorferi* enzootic cycle and encodes an outer membrane-associated protein

**Fig 1:**
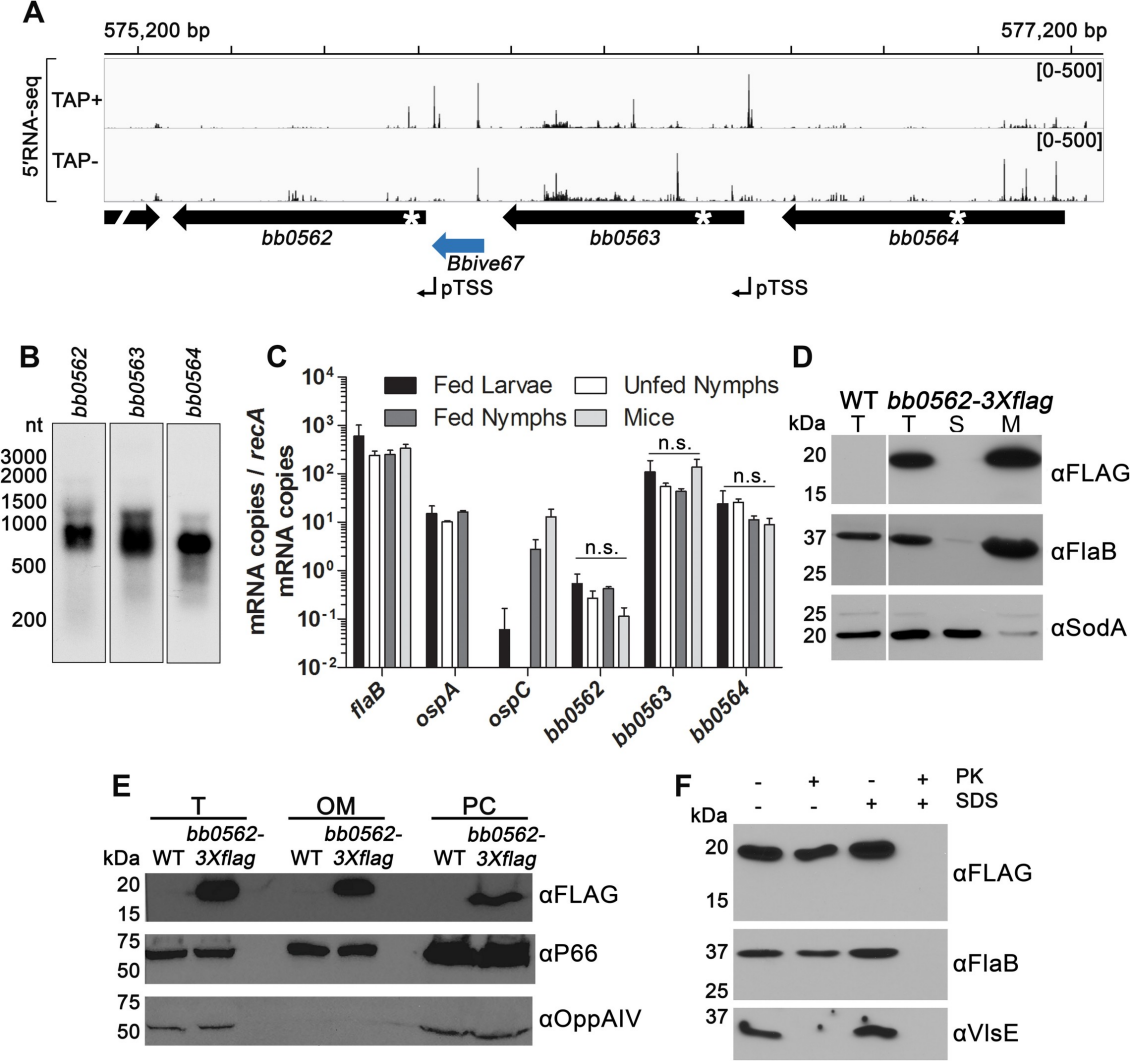
Gene *bb0562* is constitutively expressed throughout the enzootic cycle and produces an outer membrane-associated, non-surface exposed protein. (A) RNA sequencing browser image of the *bb0562-bb0564* locus displaying sequencing reads and identifying primary transcription start sites (pTSS) from 5′RNA-seq [28]. Tracks represent an overlay of two biological replicates. Read count ranges are shown in the top of each frame. The chromosome nucleotide coordinates, relative orientation of annotated ORFs (wide black arrows), *Bbive* sequence (wide blue arrow) [27,28], and pTSSs (bent black arrows) [28] are indicated. (B) Northern analysis of the *bb0562*, *bb0563*, and *bb0564* transcripts. Total RNA from wild-type *B*. *burgdorferi* was separated on an agarose gel, transferred to a membrane, and probed for the indicated RNAs (RNAs were probed sequentially on the same membrane). Size markers are displayed on the left, which is aligned for all blots. White asterisks in panel A indicate the approximate locations of the oligonucleotide probes. (C) Reverse transcription (RT)-qPCR analysis of *bb0562*, *bb0563* and *bb0564* expression in infected tick and mouse bladder tissue samples. Genes *flaB*, *ospA* and *ospC* served as controls [62]. Copy numbers for each gene target were normalized to *recA* mRNA copies. Data are presented as the average of biological triplicate samples ± standard deviation. Expression levels across samples for each target gene were compared by one-way ANOVA with Tukey’s multiple comparison test, GraphPad Prism 9.0.0. (n.s., not significant). (D) Total protein lysate (T) of wild-type (WT) or *B*. *burgdorferi* producing BB0562-3XFLAG (*bb0562-3Xflag*) was fractionated into the soluble (S) and membrane (M) components using ultracentrifugation. Protein fractions from equivalent numbers of spirochetes were subjected to SDS-PAGE and analyzed by immunoblot with antibodies against BB0562-3XFLAG (αFLAG), membrane-associated FlaB (αFlaB), or cytoplasmic SodA (αSodA). Molecular weights are shown in kilodaltons (kDa). (E) Total protein lysate (T) of wild-type (WT) or *B*. *burgdorferi* producing BB0562-3XFLAG (*bb0562-3Xflag*) was fractionated into the outer membrane (OM) and protoplasmic cylinder (PC) components by discontinuous sucrose gradient. Protein fractions from equivalent numbers of spirochetes were subjected to SDS-PAGE and analyzed by immunoblot with antibodies against BB0562-3XFLAG (αFLAG), OM-associated P66 (αP66), or PC-associated OppAIV (αOppAIV). Molecular weights are shown in kilodaltons (kDa). (F) Equal numbers of *B*. *burgdorferi* producing BB0562-3XFLAG were either treated with (+) or without (-) 200 μg/ml proteinase K (PK) in the presence (+) or absence (-) of 0.1% SDS. Samples were subjected to SDS-PAGE and analyzed by immunoblot with antibodies against BB0562-3XFLAG (αFLAG), periplasmic-associated FlaB (αFlaB), or outer surface-associated VlsE (αVlsE). Protein standards in kilodaltons (kDa) are indicated.

*In vivo* expression technology for *B*. *burgdorferi* (BbIVET) identified an infection-active putative promoter sequence immediately upstream of gene *bb0562* [27,28]. Furthermore, using global 5′ end RNA-seq analysis we detected a primary transcription start site (pTSS) for *bb0562* located at the 3′ end of the BbIVET-identified sequence (Fig 1A) [28]. Gene *bb0562* is annotated to encode a 180 amino acid (aa) hypothetical protein of unknown function and to be comprised of the conserved domain of unknown function DUF3996 (pfam13161) [29]. DUF3996 is also found across adjacent encoded proteins BB0563 (172 aa) and BB0564 (201 aa), raising the possibility of shared function between the three proteins. BB0562, BB0563 and BB0564 each demonstrate high amino acid identity within *Borrelia burgdorferi* sensu stricto isolates (97–99%) and across the *Borrelia burgdorferi* sensu lato complex (78–97%). Despite containing the same conserved domain, which spans the majority of all three proteins, the proteins share limited amino acid identity with each other (24–29%). Genes *bb0562* and *bb0563* are separated by 164 bps and genes *bb0563* and *bb0564* by 82 bps. We previously identified a pTSS for *bb0563* but not *bb0564* [28] (Fig 1A). Northern blot analysis revealed primarily monocistronic transcripts for each of the three genes (Fig 1B). Furthermore, genes *bb0562*, *bb0563* and *bb0564* were found to be constitutively expressed across all stages of the tick-mouse enzootic cycle, though the overall levels of *bb0562* were lower than that of *bb0563* and *bb0564* (Fig 1C).

Bioinformatics analysis suggests that BB0562 may harbor multiple transmembrane domains [30–32]. Furthermore, proteomic analysis of spirochete membranes has suggested that BB0562 is membrane associated in *B*. *burgdorferi* (strain B31) [33], *B*. *afzelii* (strain K78), and *B*. *garinii* (strain PBi) [34]. To experimentally test BB0562 protein production and localization in the spirochete, the sequence for a triple flag epitope (3X*flag*) was added to the C-terminus of *bb0562*. Western blot analysis of total protein lysate from the *bb0562*-3X*flag* clone using anti-FLAG antibodies specifically detected a ~20 kDa protein, the predicted molecular mass of BB0562 (Fig 1D). BB0562-3XFLAG, like membrane-associated FlaB but unlike soluble SodA, was found to be restricted to the spirochete membrane fraction (Fig 1D). To further dissect BB0562 localization, outer membrane and protoplasmic cylinder fractions were collected. Similar to outer membrane protein P66, but in contrast to inner membrane protein OppAIV, BB0562-3XFLAG was detected in both fractions, suggesting that BB0562 is associated with the outer membrane (Fig 1E). Proteinase K treatment of intact *bb0562*-3X*flag* spirochetes did not affect the amount or size of BB0562-3XFLAG (Fig 1F). Similarly, the periplasmic FlaB protein was unaffected by proteinase K treatment, whereas this treatment resulted in complete loss of the known surface exposed outer membrane protein VlsE. In contrast, BB0562-3XFLAG was susceptible to proteolysis upon permeabilization of the spirochetes with SDS (Fig 1F). Together these data support a model in which BB0562 is associated with the outer membrane or a protein(s) on the inner side of the outer membrane and is likely not a transmembrane outer membrane protein or surface exposed.

### Gene *bb0562* provides a physiological function important for *B*. *burgdorferi* disseminated infection

**Fig 2:**
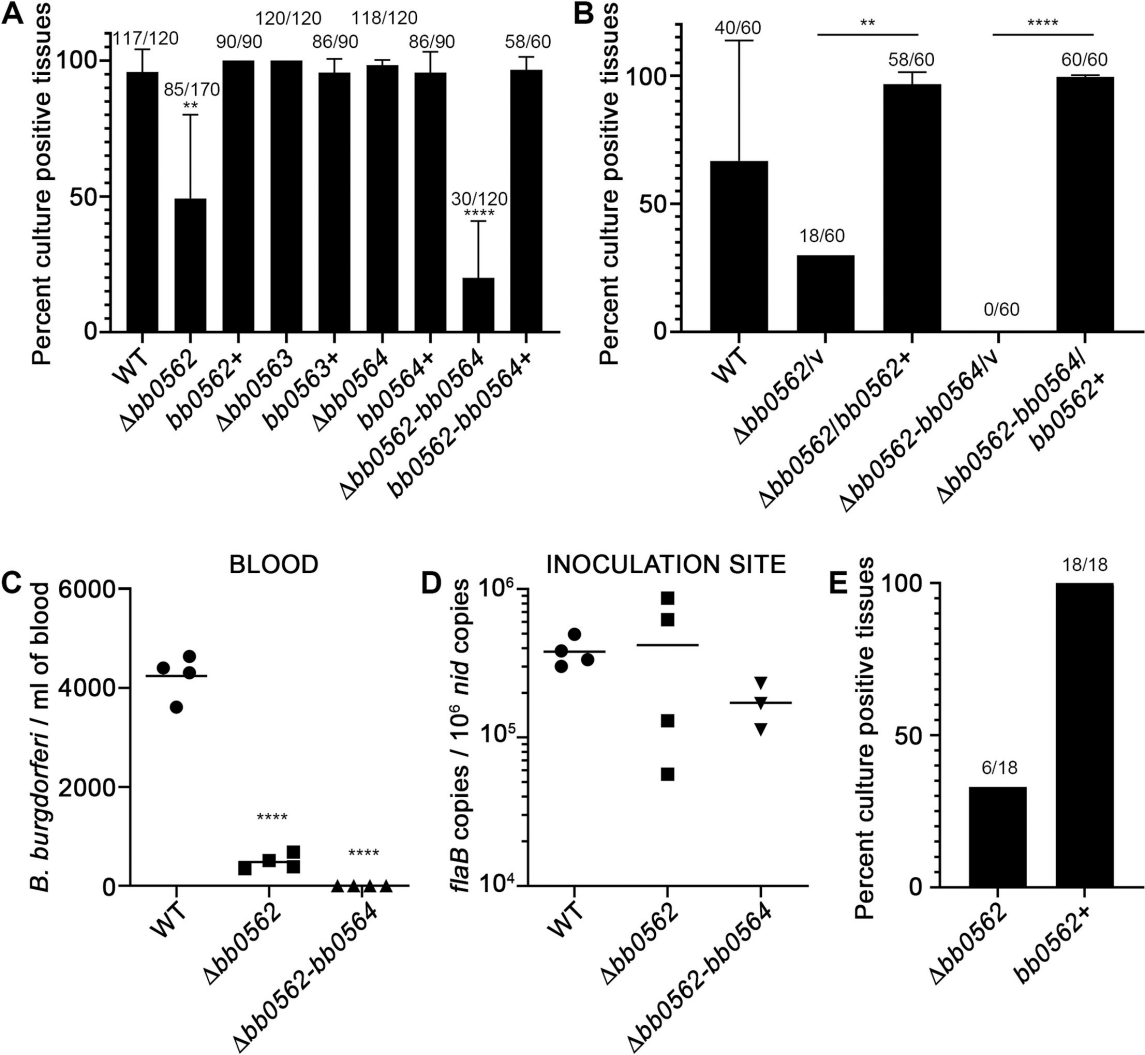
Gene *bb0562* is important for optimal disseminated infection in both immunocompetent and immunocompromised mice. Groups of 4–6 immunocompetent C3H/HeN mice were needle inoculated intradermally with 10^4^ *B*. *burgdorferi* per clone. (A and B) Mice were assessed for infection three weeks post-inoculation. Distal tissues were collected and analyzed for spirochete reisolation in culture medium. Data are presented as the average percent culture positive tissues ± standard deviation and represent 2–4 biological replicates. Numbers above each data column represent the total number of culture positive tissues out of the total number of tissues analyzed. (C) Six days post-inoculation, blood was collected and assessed for disseminating spirochetes by limiting dilution in liquid medium in 96-well plates. Each data point represents an individual mouse. The mean is indicated by a horizontal black line. (D) Seven days post-inoculation, total DNA was extracted from the skin inoculation sites. *B*. *burgdorferi* load was measured by quantifying *B*. *burgdorferi flaB* copies normalized to 10^6^ mouse *nid* copies using quantitative PCR. Each data point represents an individual mouse. The mean is indicated by a horizontal black line. (E) Groups of 6 immunocompromised NSG mice were needle inoculated intradermally with 10^4^ *B*. *burgdorferi* per clone and assessed for infection three weeks post-inoculation as described in A and B. Statistical analyses were performed using the one-way ANOVA with Dunnett’s multiple comparisons test to WT (A, C and D) or unpaired t-test (B), GraphPad Prism 9.0.0. (**p<0.01, ****p<0.0001).

In order to assess the contributions of *bb0562*, *bb0563* and *bb0564* to *B*. *burgdorferi* biology, targeted deletion of each gene individually or all three together was carried out by allelic exchange and each deletion was genetically complemented by restoration of a wild-type copy of the gene(s) in the mutant locus (S1A Fig). The mutant and complement clones were verified by end-point PCR (S1B Fig) and RT-qPCR analysis (S2 Fig). For the most part, expression levels of adjacent genes were unaltered in the mutant clones. Yet, expression of *bb0563* and *bb0564* tended to be reduced in the absence of *bb0562* (S2 Fig). In the complement clones, wild-type or near wild-type levels of expression were restored for each of the target genes, with the exception of the amounts of expression of *bb0562* and *bb0564* in *bb0562*-*bb0564*+ *B*. *burgdorferi*, which were significantly reduced compared to that of wild-type (S2 Fig). No defect was detected for any of the mutants during growth in complete BSKII liquid medium (S3 Fig) and no obvious morphological alterations or motility defects were observed under routine dark field microscopy. Groups of mice were needle inoculated with 10^4^ of each of the *B*. *burgdorferi* mutant and complement clones and assessed for infection after 3 weeks by reisolation of spirochetes from tissues. *B*. *burgdorferi* clones lacking *bb0562* (Δ*bb0562* and Δ*bb0562*-*bb0564*) demonstrated attenuated disseminated infection with an average of 49% and 20% of tissues positive for spirochete reisolation, respectively, compared to 96–100% for the *B*. *burgdorferi* clones carrying *bb0562* (Fig 2A and S2 Table). Genes *bb0563* and *bb0564* alone were found to be dispensable for mouse infection. Although these data suggested that the triple mutant may be more highly attenuated than the single Δ*bb0562* mutant, the infectivity defect of Δ*bb0562*-*bb0564 B*. *burgdorferi* was fully restored with the reintroduction of *bb0562* alone *in trans* on the shuttle vector pBSV2G (Δ*bb0562-bb0564*/*bb0562*+) as assessed by reisolation of spirochetes from tissues (Fig 2B) and quantification of spirochete loads in tissues by quantitative PCR (S4 Fig), indicating that of genes *bb0562-bb0564*, *bb0562* was sufficient for optimal infection.

Productive disseminated infection relies on successful spirochete population expansion in the skin site of infection and hematogenous spread to the tissues [35]. To evaluate the contributions of gene *bb0562* to early steps in dissemination, spirochete burdens were quantified in the blood and skin site of inoculation at days 6 and 7 post-inoculation, respectively. Strikingly, the numbers of Δ*bb0562* and Δ*bb0562*-*bb0564 B*. *burgdorferi* in the blood were significantly reduced compared to that of wild-type *B*. *burgdorferi* (Fig 2C). In contrast, there was no *bb0562*-dependent defect observed for *B*. *burgdorferi* loads in the skin inoculation site (Fig 2D). These data suggested that *bb0562* was dispensable for localized expansion of *B*. *burgdorferi* in the skin but important for spirochete survival and dissemination in the blood.

To gain further insight into the potential barriers to Δ*bb0562 B*. *burgdorferi* infectivity, we examined the ability of the mutant to infect immunodeficient mice. NOD-*scid* IL2Rgamma^null^ (NSG) mice lack key components of innate immunity and the entire adaptive immune system [36]. The Δ*bb0562 B*. *burgdorferi* mutant was defective for infection of NSG mice and resulted in only 33% of tissues positive for spirochete reisolation (Fig 2E and S2 Table). All tissues from NSG mice inoculated with *bb0562*^+^ *B*. *burgdorferi* were positive for spirochete reisolation (Fig 2E and S2 Table). These data indicated that the immunocompromised state of the NSG mice was not sufficient to rescue the infectivity defect of Δ*bb0562* spirochetes and suggested that the molecular function of BB0562 was likely to be metabolic in nature rather than a mechanism of immune evasion.

### Gene *bb0562* contributes to *B*. *burgdorferi* murine infection by tick bite

**Fig 3:**
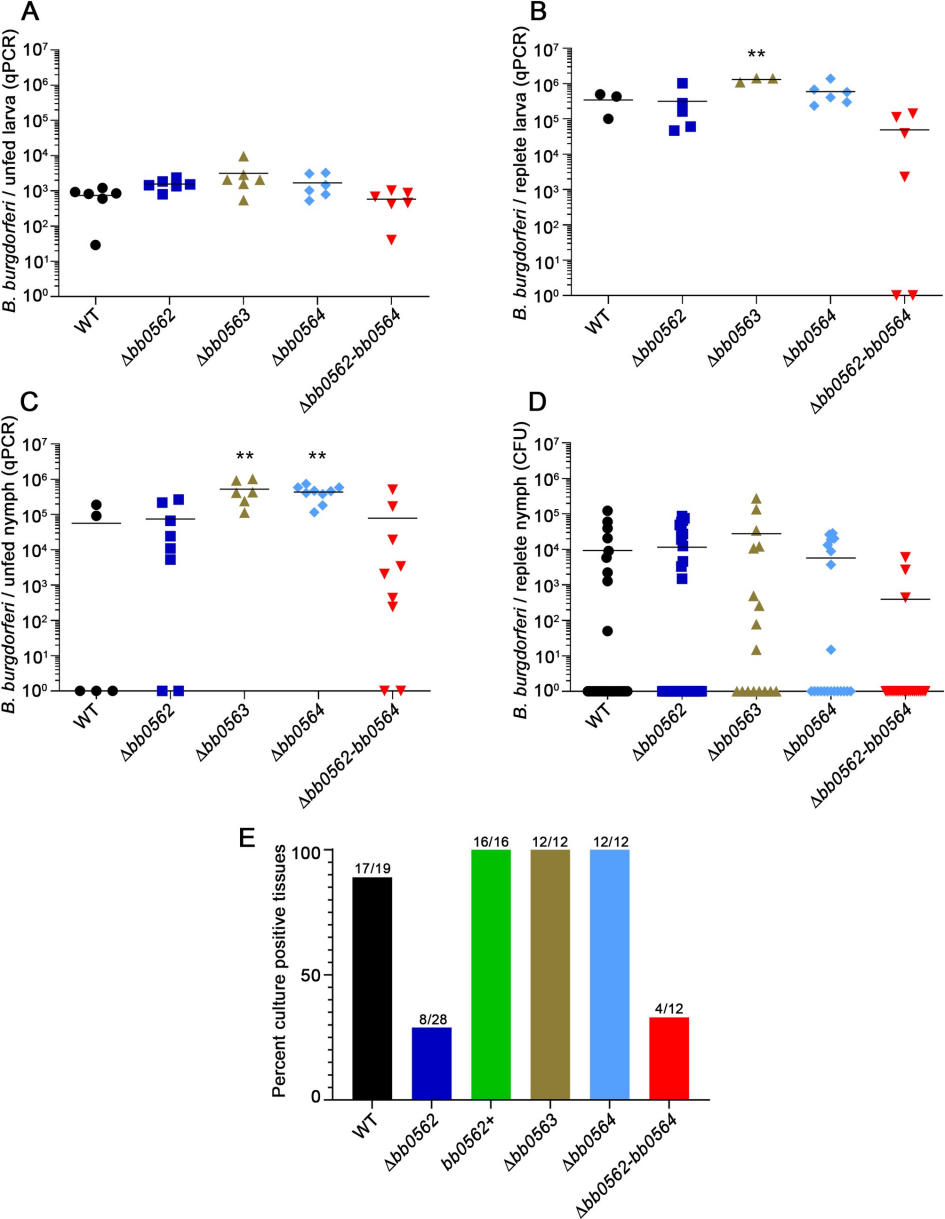
Genes *bb0562*-*bb0564* are dispensable for *B*. *burgdorferi* survival and replication in ticks but *bb0562* is important for mouse infection by tick bite transmission. Naïve *Ixodes scapularis* larval ticks were artificially infected with *B*. *burgdorferi* clones by immersion. *B*. *burgdorferi* densities in ticks were assessed at each stage of tick development before and after feeding to repletion on mice by performing quantitative PCR analysis of total DNA isolated from ticks (A-C) or plating of dilutions of whole triturated ticks in solid or liquid BSK medium (D). Statistical analyses were performed using the one-way ANOVA with Dunnett’s multiple comparisons test to WT, GraphPad Prism 9.0.0. (**p<0.01). (E) Cohorts of 25 *B*. *burgdorferi* infected nymphs were fed to repletion on groups of 3–7 naïve C3H/HeN mice per *B*. *burgdorferi* clone. Mice were assessed for infection three weeks post-tick feeding. Ear, heart, bladder, and joint tissues were collected and analyzed for spirochete reisolation in culture medium. Numbers above each data column represent the total number of culture positive tissues out of the total number of tissues analyzed.

Due to the attenuation of Δ*bb0562 B*. *burgdorferi* for infection of both immunocompetent and immunocompromised mice by needle inoculation, we were interested in determining the contribution of *bb0562* to *B*. *burgdorferi* survival throughout the tick-mouse infectious cycle. Naïve *Ixodes scapularis* larvae were artificially infected with the *B*. *burgdorferi* clones. All clones colonized the unfed larvae with the same efficiency (Fig 3A). Moreover, no defect in spirochete load was detected at any tick life stage for any of the mutants (Fig 3B and 3D). Compared to wild-type *B*. *burgdorferi*, a significant increase in the number of spirochetes was detected for Δ*bb0563 B*. *burgdorferi* and Δ*bb0564 B*. *burgdorferi* in replete larvae and/or unfed nymphs, respectively. Yet, there were no statistical differences in spirochete number in fed nymphs for any of the mutants compared to that of wild-type *B*. *burgdorferi*. Overall, these data suggested that genes *bb0562-bb0564* were dispensable for *B*. *burgdorferi* survival and replication in ticks. However, consistent with the needle inoculation studies, *bb0562* was found to be important for mouse infection by tick bite. Mice fed on by Δ*bb0562*-infected nymphs demonstrated low infection rates, as determined by spirochete reisolation from tissues, compared to mice fed on by *bb0562*^+^-infected ticks (Fig 3E and S2 Table). These data further supported a significant role for *bb0562* in *B*. *burgdorferi* mammalian infectivity.

### *bb0562* is critical for *B*. *burgdorferi* growth in fatty acid limited conditions

**Fig 4:**
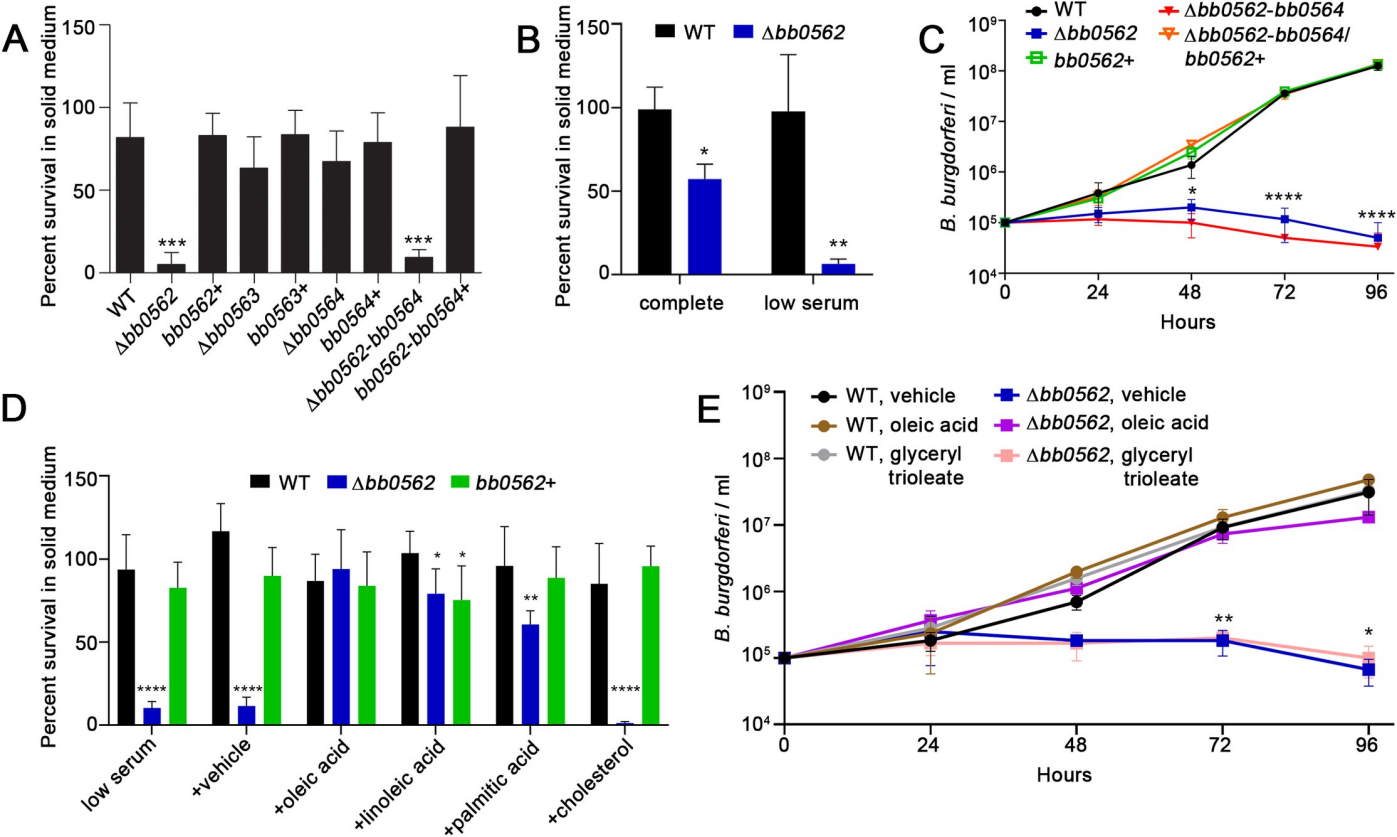
Gene *bb0562* is important for *B*. *burgdorferi* growth in media lacking free fatty acids. (A) Triplicate cultures of each *B*. *burgdorferi* clone were subcultured at 1x10^5^ cells/ml and grown in complete BSKII liquid medium. At the 48-hour time point an aliquot of each culture was removed, and dilutions plated in complete solid medium and by limiting dilution in complete liquid medium in 96-well plates. Data are presented as percent survival in solid medium defined as the percent survival in solid medium [(actual cfu/expected cfu) x 100] normalized to the percent survival in liquid medium [(actual positive wells/expected positive wells) x 100]. (B) At the 72-hour time point an aliquot of each culture was removed, and dilutions plated in complete or 1.8% serum (low serum) solid medium and by limiting dilution in complete liquid medium in 96-well plates. Data are presented as percent survival in solid medium. (C) Triplicate cultures of each *B*. *burgdorferi* clone were grown in liquid medium containing 1.8% serum over 96 hours and the densities of the cultures were determined via Petroff-Hausser count under dark field microscopy every 24 hours. (D) Triplicate cultures of each *B*. *burgdorferi* clone were grown in complete liquid medium. At the 72-hour time point an aliquot of each culture was removed, and dilutions plated in 1.8% serum (low serum) solid medium alone or with the addition of 0.7% ethanol (vehicle), 20 μg/ml oleic acid, 20 μg/ml linoleic acid, 20 μg/ml palmitic acid, or 20 μg/ml cholesterol and by limiting dilution in complete liquid medium in 96-well plates. Data are presented as percent survival in solid medium. (E) Triplicate cultures of each *B*. *burgdorferi* clone were grown in liquid medium containing 1.8% serum plus 0.7% ethanol (vehicle), 20 μg/ml oleic acid or 20 μg/ml glyceryl trioleate over 96 hours and the densities of the cultures were determined via Petroff-Hausser count under dark field microscopy every 24 hours. All data represent the average ± standard deviation. Statistical analyses were performed using the one-way ANOVA with Dunnett’s multiple comparisons test to WT (A and C-E) or unpaired t-test (B), GraphPad Prism 9.0.0. (*p<0.05, **p<0.01, ***p<0.001, ****p<0.0001).

Although we did not detect a growth phenotype for any of the mutants in complete BSKII liquid medium (S3 and S5A Figs), over the course of our studies we observed a variable defect in the growth of Δ*bb0562* and Δ*bb0562*-*bb0564 B*. *burgdorferi* in complete solid BSK medium. To explore this observation further, aliquots of spirochetes collected at different time points across a 166-hour growth curve (S5A Fig) were quantified for colony forming units in both solid medium and liquid medium in a 96-well plate format. The data were reported as the percent survival in solid medium, which represented the number of colonies in solid medium normalized to the number of colonies by limiting dilution in complete liquid medium in 96-well plates (see Materials and Methods for more details). At mid-log phase (48-hour time point) Δ*bb0562* and Δ*bb0562*-*bb0564 B*. *burgdorferi* both demonstrated significant survival defects in solid medium relative to wild-type *B*. *burgdorferi*, whereas the mutant clones harboring *bb0562* did not (Fig 4A). The defects of the *bb0562* mutant clones were the most pronounced for spirochetes in mid-log and early stationary phase (S5 Fig). Recently, bacterial colony formation in solid media has been linked to fatty acid availability [37]. Because the *B*. *burgdorferi* genome lacks the genes for de novo synthesis of fatty acids [9], we hypothesized that *bb0562* may contribute to *B*. *burgdorferi*’s ability to scavenge fatty acids from the growth environment. Furthermore, we reasoned that rabbit serum likely provides the major source of fatty acids in *Borrelia* media and the concentration of fatty acids in rabbit serum could vary from lot to lot leading to the variation in the growth defect that we observed. Consistent with this notion, reduction in the amount of rabbit serum in solid BSK medium from 4% (complete) to 1.8% (low serum) resulted in a decreased percent survival of the Δ*bb0562* mutant relative to the wild-type clone in the same condition (Fig 4B). Interestingly, the *bb0562*-dependent growth defect in low serum was not limited to growth in solid medium. This phenotype was also detected for Δ*bb0562* and Δ*bb0562*-*bb0564 B*. *burgdorferi* grown in liquid medium containing only 1.8% serum (Fig 4C). Similar to the mouse infection defect of Δ*bb0562*-*bb0564 B*. *burgdorferi*, reintroduction of *bb0562* alone to the triple mutant was sufficient to restore wild-type levels of growth in low serum medium (Fig 4C). The long chain monounsaturated fatty acid, oleic acid (c18:1n9), long chain polyunsaturated fatty acid, linoleic acid (c18:2n6), and the saturated fatty acid, palmitic acid (c16:0), have been shown to comprise the vast majority of the fatty acid composition of *B*. *burgdorferi* grown in BSK media [38]. Each of these three fatty acids, or cholesterol, a significant non-fatty acid sterol lipid present in the *B*. *burgdorferi* membrane [21], was added to the low serum medium and the *B*. *burgdorferi* clones were assessed for growth. Strikingly, addition of oleic acid fully restored the *bb0562*-dependent growth defect in both low serum solid and liquid media to that of wild-type (Fig 4D and 4E). Similarly, a partial rescue of Δ*bb0562 B*. *burgdorferi* growth in low serum solid medium occurred with the addition of linoleic acid or palmitic acid, but not with the addition of cholesterol (Fig 4D), together supporting the fatty acid dependence of the phenotype. In mammals, fatty acids are primarily stored in fat droplets in the form of triglycerides [39]. To model this, we examined the ability of the *B*. *burgdorferi* clones lacking *bb0562* to grow in low serum liquid medium supplemented with the triglyceride glyceryl trioleate, which is comprised of three oleic acid molecules and a glycerol core. Unlike free oleic acid, glyceryl trioleate had no effect on the growth defect of Δ*bb0562 B*. *burgdorferi* (Fig 4E), suggesting that free fatty acids are required to support the growth of the mutant and that *bb0562* may be important for release of fatty acids from triglycerides for spirochete growth.

### BB0562 is a lipolytic enzyme

**Fig 5:**
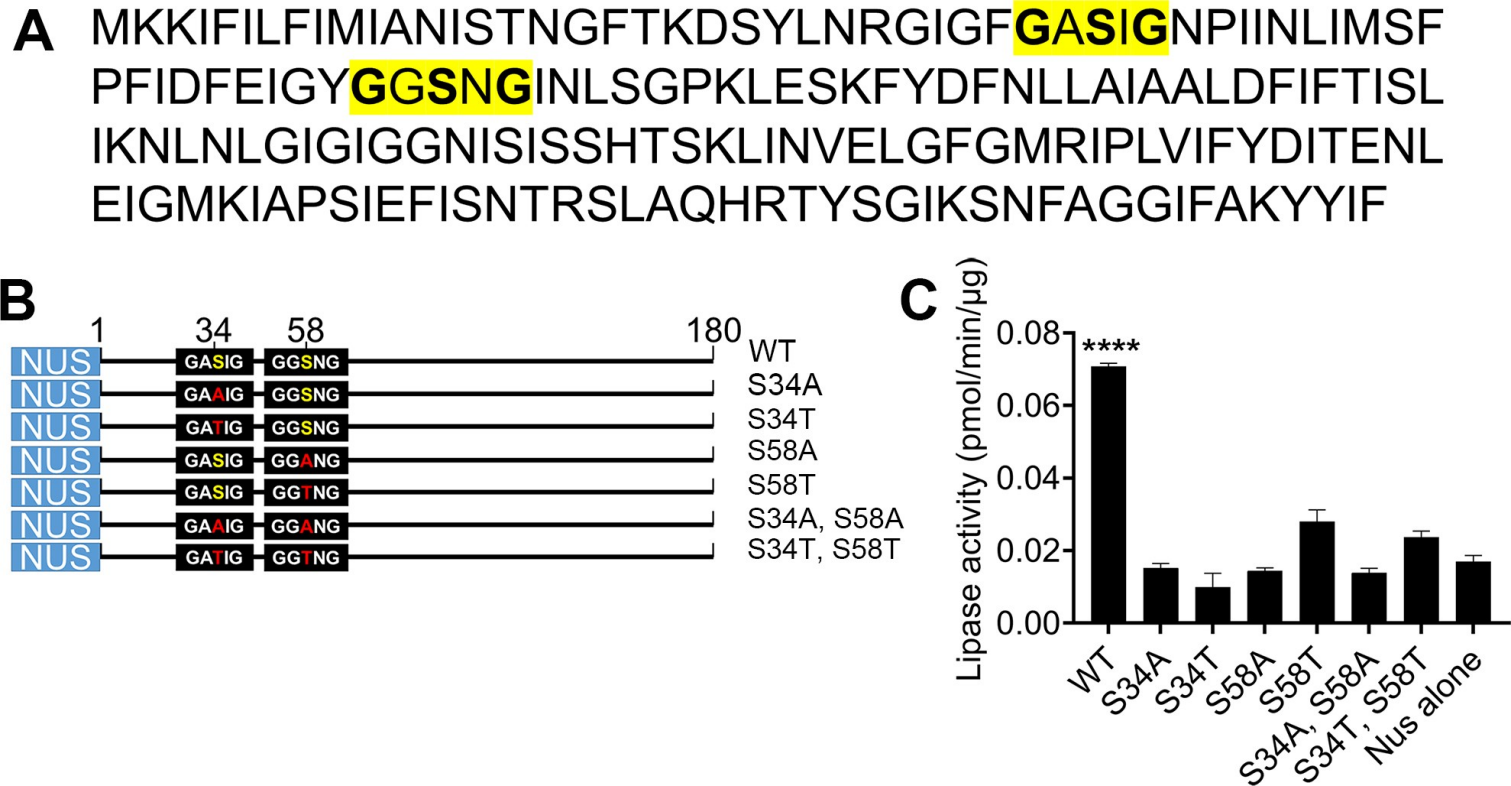
BB0562 demonstrates lipolytic activity dependent on two canonical GXSXG motifs. (A) Primary amino acid sequence of BB0562. The two canonical GXSXG motifs of lipase/esterase enzymes are highlighted in yellow. (B) Schematic representation of the recombinant Nus-BB0562 proteins. The putative catalytic serine residues, S34 and S58, within the GXSXG motifs, as well as the alanine and threonine mutations at those sites are shown. Numbers indicate amino acid positions. (C) The lipase activities of purified recombinant wild-type (WT) Nus-BB0562, single and double mutant proteins and Nus alone were calculated as picomoles (pmol) of methylresorufin produced from hydrolysis of a long chain fatty acid triglyceride derivative substrate per minute (min) per microgram (μg) of recombinant protein (pmol/min/μg). Error bars represent standard deviation from the mean of triplicate measures. Statistical analyses were performed using the one-way ANOVA with Dunnett’s multiple comparisons test to WT, GraphPad Prism 9.0.0 (****p<0.0001).

Triacylglycerol lipases target hydrolysis of triacylglycerol to glycerol and free fatty acid [40] and typically have an α/β hydrolase fold and contain the active-site consensus motif GXSXG or GDSL [41,42]. Although the BB0562 protein lacks both overall primary sequence and tertiary structural homology to known bacterial lipases, visual inspection of the BB0562 amino acid sequence revealed two GXSXG motifs, with putative catalytic serine residues at amino acids 34 and 58 (Fig 5A and 5B). No lipase motifs were found in the amino acid sequences of BB0563 or BB0564. Recombinant BB0562 was produced in *E*. *coli* and purified as an N-terminal fusion to the highly soluble Nus protein along with N- and C-terminal 6XHis tags (S6 Fig). In addition, we generated mutant BB0562 proteins in which the putative catalytic serine residues alone or together were changed to alanine(s) or threonine(s) (Fig 5B). All seven recombinant proteins, along with the Nus protein alone, were examined for lipolytic activity in a fluorometric lipase assay. Strikingly, wild-type BB0562 demonstrated lipase activity significantly greater than that of the serine mutants and Nus alone (Fig 5C). These data indicate that BB0562 is a lipolytic enzyme whose activity is dependent on the serine residues in two GXSXG catalytic motifs.

## Discussion

*B*. *burgdorferi* is an obligate pathogen, dependent on its host environments for essential nutrients for survival. Therefore, nutrient scavenging mechanisms are critical to *B*. *burgdorferi* infection and pathogenesis. BbIVET identified numerous *B*. *burgdorferi* genes of unknown function that are expressed during mouse infection, including *bb0562*, which we have demonstrated encodes a novel lipase that promotes *B*. *burgdorferi* survival in environments low in free fatty acids, including the mammalian host. BB0562 adds to the arsenal of nutritional virulence determinants of *B*. *burgdorferi* important for the pathogen to be maintained in the tick-mouse enzootic cycle and to cause disseminated disease.

Lipolytic enzymes are broadly present across pathogenic and nonpathogenic bacterial species [40,42]. A number of pathogens rely on lipases to hydrolyze host lipids for release of fatty acids for nutrients and several of these enzymes have also been shown to be important for infection. For example, *Mycobacterium tuberculosis* virulence, persistence, and drug-resistance have been linked to lipid acquisition and metabolism [43]. *M*. *tuberculosis* is predicted to possess up to 30 lipolytic enzymes [44,45], some of which contribute to virulence mechanisms such as intracellular growth, bacterial loads in mice and recovery from dormancy [46–50]. *Pseudomonas aeruginosa* lipases, LipA and LipC, influence motility and biofilm formation as well as the production of virulence factors pyoverdine and rhamnolipid [51,52]. Similarly, staphylococcal lipases play roles in biofilm formation and host cell invasion [53]. Moreover, phospholipases of numerous pathogenic bacteria, which target phospholipids in eukaryotic membranes, have been associated with cytotoxicity, host cell invasion, tissue colonization, hemolytic activity, phagosome escape and necrosis [54]. In total bacterial lipases are key components of host-pathogen interactions whose activities have important consequences for survival, dissemination, and pathogenesis.

Gene *bb0562* and adjacent genes *bb0563* and *bb0564*, were all predicted to encode outer membrane proteins containing the conserved domain of unknown function, DUF3996, unique to *Borrelia* species [9,29], suggesting they may confer similar functions. DUF3996 is also present across two other *B*. *burgdorferi* proteins, BB0405 (203 aa) and BB0406 (203 aa) [33,55–57]. The *bb0405* and *bb0406* genes are co-transcribed, expressed throughout the enzootic cycle and encode immunogenic outer membrane proteins [33,55]. Gene *bb0405* is required for *B*. *burgdorferi* infection but *bb0406* is not [56,57]. The BB0405 homolog in *B*. *afzelii*, BAPKO0422, has been shown to be a factor H binding protein, suggesting a role in complement evasion [58]. Northern blot analysis documented primarily monocistronic transcripts for *bb0562*, *bb0563* and *bb0564*, though longer faint bands were also detected. Given that the RNA was isolated from culture-derived *B*. *burgdorferi*, it remains a possibility that some environmental/stress conditions could favor the production of a polycistronic transcript(s). Nevertheless, we did not observe differential expression of any of the three genes throughout the tick-mouse infection model, consistent with other studies [59–62]. Only gene *bb0562*, and not *bb0563* or *bb0564*, was found to be important for *B*. *burgdorferi* infection and fatty acid-dependent survival, suggesting *bb0563* and *bb0564* may have disparate roles from *bb0562*. Furthermore, the lipase function of BB0562 appears to be independent of DUF3996. The catalytic lipase motifs are only present in BB0562 and not the other DUF3996 members. Future work is needed to understand the link, if any, between the five DUF3996 proteins.

Initial characterization of *bb0562* began with identification of the contribution of the gene to mouse infection. By both needle inoculation and tick bite transmission *bb0562* was important for maximal disseminated infection. Moreover, reintroduction of *bb0562* alone to *B*. *burgdorferi* lacking *bb0562*, *bb0563* and *bb0564* was sufficient to restore wild-type levels of infection. The dissemination defect of *B*. *burgdorferi* lacking *bb0562* was traced to substantially reduced survival in the blood. The *bb0562*-dependent infection defect was not ameliorated in immunocompromised mice, suggesting that *bb0562* provides a physiological rather than an immune evasion-related function. Combined with the observation of a growth defect of Δ*bb0562* spirochetes in solid BSK medium, these data led to the hypothesis that *bb0562* is important for *B*. *burgdorferi* growth and survival in environments deplete in free fatty acids. We found that the rabbit serum component of the *in vitro* growth media provides the critical source of fatty acids for *B*. *burgdorferi*, a fatty acid auxotroph, to scavenge. This growth defect was discovered in solid medium, likely because solid BSK medium is supplemented with 4% rabbit serum, whereas liquid BSKII medium contains 6% rabbit serum, which appears to supply a sufficient amount of free fatty acids for spirochete growth in the absence of *bb0562*. *E*. *coli* lacking the *fabA* gene, which is essential for biosynthesis of long chain unsaturated fatty acids, is highly attenuated for colony formation on solid media under fatty acid limited conditions [37]. However, this phenotype is less pronounced for growth in liquid media, suggesting that fatty acids are particularly important for bacterial colony formation *in vitro* [37].

The *bb0562*-dependent growth defect in low serum media was fully overcome by supplementation with the long chain monounsaturated fatty acid oleic acid. Partial rescue was observed with the addition of the long chain polyunsaturated fatty acid linoleic acid or the long chain saturated fatty acid palmitic acid. These three fatty acids have been shown to be the major fatty acid constituents of the *B*. *burgdorferi* membrane when the spirochete is grown in BSK media [19,20,22]. Addition of cholesterol, a non-fatty acid sterol lipid constituent of the *B*. *burgdorferi* membrane [21], did not rescue the growth defect of spirochetes lacking *bb0562*, indicating that the phenotype was fatty acid specific. Strikingly, oleic acid, when provided in the form of a triglyceride, was not able to support the growth of *B*. *burgdorferi* lacking *bb0562*, which led to identification of BB0562 as a novel *B*. *burgdorferi* lipase. Indeed, purified, recombinant BB0562 demonstrated lipase activity, which was dependent on the serine residues in the two canonical GXSXG lipase motifs identified in the protein. In bacteria, lipases have been predominantly shown to be secreted extracellularly [40,42] and there are some examples of lipases that localize to the bacterial outer surface [63,64], both of which likely allow interaction of the lipase with target substrates in the environment. Our data indicate that BB0562 is associated with the spirochete outer membrane but is likely not surface exposed. Given this, the mechanism for how the protein interacts with host molecules for fatty acid scavenge remains unknown and warrants further study.

Bacterial lipases are currently classified into 19 families based on amino acid sequence and physiological properties [42]. BB0562 was unrecognized as a putative lipase by conserved domain analysis and rather was annotated as a hypothetical protein of unknown function [29,65,66]. The findings of our functional analysis of Δ*bb0562 B*. *burgdorferi* led us to analyze the amino acid sequence of the protein more closely resulting in our identification of two canonical GXSXG lipase motifs and characterization of the lipolytic activity of the protein. Mutational analysis of the serine residue in each GXSXG motif demonstrated that the enzymatic activity of the protein was dependent on both active sites. Despite the wide diversity of bacterial lipolytic enzymes, to our knowledge CT149, a cholesterol esterase of *Chlamydia trachomatis*, is the only other protein that harbors two GXSXG motifs [67]. It is interesting to note that serine 34 is conserved across the BB0562 homologs of Lyme disease and Relapsing Fever spirochetes, however, serine 58, while conserved across the BB0562 homologs of Lyme disease spirochetes, is a threonine residue in the homologs of Relapsing Fever spirochetes. It is possible that these active site motif differences result in substrate specificity differences that reflect distinct nutritional needs of the two groups of pathogenic spirochetes. In addition, the apparent higher lipase activities of the recombinant Nus-BB0562 proteins carrying the S58T mutation relative to the other mutant proteins tested, perhaps suggests the ability of a threonine residue in this position to confer some enzymatic activity. BB0562 has a theoretical molecular mass of 19.8 kDa and lacks any cysteine residues, two characteristics of triacylglycerol lipase subfamily I.4, which is comprised of the smallest triacylglycerol lipases primarily from *Bacillus* species [42]. Future work is aimed at biochemical characterization of BB0562 to define the breadth of substrate specificity and function relative to other known lipases.

The lipase activity of BB0562 plays a key role in fatty acid acquisition by *B*. *burgdorferi* during infection of the mammalian host, likely via the release of fatty acids from host triglycerides and/or chylomicrons. This model is supported by the significant growth defect of spirochete lacking *bb0562* in the blood but not the inoculation site skin tissue. Lipid concentration analysis of mouse tissues and blood has found significantly higher levels of free fatty acids in subcutaneous tissue compared to the amount of free fatty acids in the blood. Moreover, triglyceride concentrations in the blood have been found to be higher than that of free fatty acids [68], suggesting that the blood represents an environment in which the activity of BB0562 may be key for *B*. *burgdorferi* survival. No *bb0562-*dependent growth or survival defect was found for *B*. *burgdorferi* in ticks. Free fatty acids are likely released from the blood meal during tick feeding and digestion, bypassing the contribution of the lipase activity of BB0562 for this purpose.

Gene *bb0562* is not essential, as loss of *bb0562* did not result in absolute lethality of the spirochetes in low fatty acid *in vitro* or *in vivo* environments. The survival of *B*. *burgdorferi* in the absence of *bb0562* may be supported by the lipase activity conferred by other lipases, such as BB0646 [25]. BB0646 harbors a canonical α/β hydrolase fold and a single GXSXG lipase motif [25]. In contrast to BB0562 which appears to be important for scavenge of monounsaturated fatty acids, BB0646 demonstrates specificity for polyunsaturated and saturated fatty acid substrates [25]. In addition, BB0646 exhibits hemolytic activity, which could provide a means for *B*. *burgdorferi* to acquire polyunsaturated fatty acids from the phospholipids of host cells rather than triglycerides. Therefore, BB0562 and BB0646 may work in concert to scavenge the full complement of fatty acids required by *B*. *burgdorferi* for optimal fitness throughout the enzootic cycle. Although it remains a possibility that the lipase activity of BB0562 also contributes to an immunomodulatory function, as has been demonstrated for other pathogens [69], the finding that Δ*bb0562 B*. *burgdorferi* remained attenuated in highly immunocompromised mice suggested that BB0562 primarily functions in nutrient scavenge.

In all, this work encompasses the first investigation of gene *bb0562* and has led to its identification as a nutritional virulence determinant due to its function as a lipolytic enzyme. Bb-IVET discovered numerous previously uncharacterized genes, many of which are annotated to encode hypothetical proteins unique to *Borrelia* species. Elucidation of the contributions of non-conserved proteins of unknown function, such as BB0562, to *B*. *burgdorferi* survival and infection uncovers new opportunities to gain greater understanding of the unique biology of *B*. *burgdorferi* and perhaps discover novel therapeutic targets for the treatment of Lyme disease.

## Materials and methods

### Ethics statement

The University of Central Florida is accredited by the International Association for Assessment and Accreditation of Laboratory Animal Care. Protocols for all animal experiments were prepared according to the guidelines of the National Institutes of Health, reviewed, and approved by the University of Central Florida Institutional Animal Care and Use Committee.

### Bacterial strains and growth conditions

*Borrelia* (*Borreliella*) *burgdorferi* clones used in this study were derived from clone B31 A3 and genetic manipulations used infectious low-passage clone A3-68Δ*bbe02*, herein referred to as wild-type (WT) [70]. Cultivation of spirochetes and DH5α *E*. *coli* were as previously reported [17].

### Northern blot

*B*. *burgdorferi* cells grown to 1.15 x 10^8^ cells/ml were collected by centrifugation, washed once with 1X PBS (1.54 M NaCl, 10.6 mM KH_2_PO_4_, 56.0 mM Na_2_HPO_4_, pH 7.4) and pellet snap frozen in liquid nitrogen. RNA was isolated using TRIzol (Thermo Fisher Scientific) as described previously [71]. RNA was resuspended in DEPC H_2_O and quantified using a NanoDrop (Thermo Fisher Scientific).

Agarose northern blots were performed using total wild-type *B*. *burgdorferi* RNA as described previously [28,72]. 10 μg of RNA were fractionated on a 2% NuSieve 3:1 agarose (Lonza), 1X MOPS, 2% formaldehyde gel and transferred to a Zeta-Probe GT membrane (Bio-Rad) via capillary action overnight. The RNA was crosslinked to the membranes by UV irradiation and RiboRuler High Range RNA ladders (Thermo Fisher Scientific) were marked by UV-shadowing. Membranes were blocked in ULTRAhyb-Oligo Hybridization Buffer (Ambion) and hybridized with 5′ ^32^P-end labeled oligonucleotides probes (S1 Table). After an overnight incubation, the membranes were rinsed with 2X SSC/0.1% SDS and 0.2X SSC/0.1% SDS prior to exposure on film. Blots were stripped by two 7-min incubations in boiling 0.2% SDS followed by two 7-min incubations in boiling water.

### *B*. *burgdorferi* genetic manipulations

Targeted gene deletion and *cis* complementation was carried out via allelic exchange on the *B*. *burgdorferi* chromosome. All oligonucleotides used are listed in S1 Table. Spectinomycin/streptomycin (*flaBp*-*aadA*) and gentamycin (*flgBp*-*aacC1*) resistant cassettes were Phusion-PCR amplified from *B*. *burgdorferi* shuttle vectors [15,73,74]. Allelic exchange constructs were engineered via a PCR-based overlap extension strategy [27] using Phusion-PCR and target specific oligonucleotides and PCR-generated linear DNA transformed into the appropriate *B*. *burgdorferi* clones, as described [75]. All clones were verified by PCR using oligonucleotides external to the lesion (S1 Table and S1 Fig). All clones were verified to contain the endogenous plasmid content of the parent [76,77].

Gene *bb0562* along with 145 bps of upstream sequence were amplified with and without a 3X*flag* epitope sequence [78] at the C-terminus via Phusion-PCR using *B*. *burgdorferi* A3 genomic DNA (S1 Table) and cloned into pBSV2G [74] in *E*. *coli*. The plasmids were verified by PCR, restriction enzyme digest, and Sanger sequencing, prior to transformation into Δ*bb0562* and/or Δ*bb0562*-*bb0564 B*. *burgdorferi*, as described [75].

### BB0562 localization and western blot analysis

*B*. *burgdorferi* protein lysates were separated into soluble and membrane fractions [79]. Briefly, spirochetes were grown to log phase (3-7x10^7^ cells/ml) in BSKII medium, spun at 3210 x g for 10 min, washed twice with cold HN buffer (50 mM Hepes, 50 mM NaCl, pH 7.5), and resuspended in 1 ml HN and 100 μl Halt Protease Inhibitor Cocktail (Thermo Fisher Scientific). Spirochetes were lysed via sonication and spun at 125,000 x g to collect the soluble and membrane components of the lysate. Immunoblots were performed using DYKDDDDK(FLAG)-tag mAb (0.1 μg/ml) (Genscript), mouse anti-FlaB H9724 (1:200) [80], and polyclonal rabbit anti-SodA NIH754 (1,1000) [81] antibodies.

Outer membrane (OM) and protoplasmic cylinder (PC) fractions were isolated as previously described [82,83]. Briefly, Δ*bb0562*/pBSV2G *bb0562* (WT) and Δ*bb0562*/pBSV2G *bb0562-3Xflag* (*bb0562-3Xflag*) were harvested at a density of 5 x 10^7^ spirochetes/ml by centrifugation (20 min at 5,800 x*g* at 4°C). Pellets were washed in PBS (137 mM NaCl, 2.7 mM KCl, 10 mM Na_2_HPO_4_, 1.8 mM KH_2_PO_4_, pH 7.4) with 0.1% BSA, resuspended in 25 mM citrate buffer (pH 3.2) with 0.1% BSA, then gently rocked for two hours at room temperature. Lysates were centrifuged (30 min, 20,000 x*g* at 4°C) and the resulting pellets were resuspended in cold 25 mM citrate buffer (pH 3.2) supplemented with 0.1% BSA. Supernatants were layered on a discontinuous sucrose gradient (25%, 42%, and 56% wt/wt sucrose). Gradients were centrifuged overnight at 100,000 x*g*, 4°C. OM fractions were diluted in cold PBS and centrifuged for four hours at 141,000 x*g*, 4°C. Pellets were resuspended in 25 mM citrate buffer (pH 3.2) and separated by overnight centrifugation (100,000 x*g*, 4°C) on 10–40% continuous sucrose gradients. The OM fractions were washed in cold PBS and centrifuged for four hours at 141,000 x*g*, 4°C. Pellets were resuspended in PBS with 1 mM phenylmethylsulfonyl fluoride (PMSF) and stored at -80°C. PC fractions were washed in cold PBS and centrifuged for 20 min at 10,000 x*g*, 4°C. Pellets were resuspended in cold PBS and stored at -80°C. Three biological replicates were performed. Immunoblots of whole cell lysates, OM, and PC fractions derived from 10^8^ spirochetes were performed using DYKDDDDK(FLAG)-tag mAb (0.1 μg/ml) (Genscript), rat anti-P66 (1:1000) [84], and rat anti-OppAIV (1:1000) [85] antibodies.

Proteinase K digestions were performed as described [86,87]. Briefly, 1 x 10^9^ log phase grown (3x10^7^ cells/ml) *B*. *burgdorferi* were resuspended in PBS-Mg^2+^ (137 mM NaCl, 2.7 mM KCl, 10 mM Na_2_HPO_4_, 1.8 mM KH_2_PO_4_, 5mM MgCl_2_ pH 7.4) with or without 200 μg/ml proteinase K and incubated at 20°C for 1 hr. Simultaneously, the same conditions were performed with the addition of 0.1% SDS during the incubation. Reactions were stopped with the addition of 1 mg/ml PMSF and the samples were pelleted by centrifugation at 2000 x g. Immunoblots were performed using DYKDDDDK(FLAG)-tag mAb (0.1 μg/ml) (Genscript), mouse anti-FlaB H9724 (1:200) [80], and polyclonal rabbit anti-VlsE (1:1000) (Rockland Immunochemicals) antibodies.

### Growth analysis

Growth curves were performed in biological triplicate over up to 168 hours as described [17].

### RNA extractions and RT-qPCR analysis

Isolation of RNA from *in vitro* grown log phase (3-7x10^7^ cells/ml) *B*. *burgdorferi* was performed using TRIzol reagent (Life Technologies). For isolation of RNA from infected ticks, C3H/HeN mice (Envigo) were needle inoculated with *B*. *burgdorferi* B31 A3, at a dose of 10^5^ spirochetes and infection confirmed via positive seroreactivity to *B*. *burgdorferi* lysate, as previously described [77,88]. Three weeks post inoculation, approximately 200 naïve ~5 month old *Ixodes scapularis* (Centers for Disease Control, BEI resources) per mouse were fed to repletion as previously described [89,90]. Approximately one week following feeding to repletion, triplicate groups of 25–50 fed larvae and 13–15 fed nymphs, or five weeks following feeding to repletion, triplicate groups of 25 unfed nymphs, were snap frozen in liquid nitrogen. Ticks were homogenized in ~1 ml TE buffer (10 mM Tris, 1 mM EDTA, pH 8.0) containing 0.5 mg/ml lysozyme in gentleMACS^TM^ M tubes using a gentleMACS^TM^ Dissociator, setting RNA 2 (Miltenyi Biotech). For RNA isolation from infected mouse tissue, mice were inoculated as described and 10 days post-inoculation the animals were sacrificed, and bladder tissues harvested. In triplicate groups of 3 bladders per RNA preparation, bladders were snap frozen in liquid nitrogen and homogenized as described for infected ticks. Mouse and tick samples were transferred to 1.5 ml safe-lock Eppendorf tubes, with 1% SDS and incubated at 64°C for 2 min, followed by the addition of 0.1 M NaAOc pH 5.2. RNA extraction continued using a hot phenol protocol as described previously [28,91].

Up to 50 μg of RNA was treated with 10 U DNase I (Roche) and 80 U rRNasin (Promega) at 37°C for 15 min. The RNA samples were purified as described [91]. The DNase treatment and purification were repeated twice to ensure DNA removal from samples.

The concentrations of the *in vitro* and *in vivo*-derived samples were measured using a nanodrop spectrophotometer, and 1 μg was converted to cDNA using the iScript Select cDNA synthesis kit and random primers (Bio-Rad), according to the manufacturer’s instructions. Parallel reactions without reverse transcriptase for each sample were also performed. One microliter of neat cDNA was used as template for qPCR. cDNA was generated using DNase treated RNA and probed using primers 1123+1124 (*recA*), 1875+1876 (*flaB*), 2138+2139 (*ospA*), 2203+2204 *(ospC*), 2374+2373 (*bb0561*),1715+1716 (*bb0562*), 2179+2180 (*bb0563*), and 2263+2264 (*bb0564*) (S1 Table). The mRNA copies of each target were determined using a *B*. *burgdorferi* genomic DNA standard curve (10^7^−10^4^ genome copies).

### Mouse infection by needle inoculation

All *B*. *burgdorferi* clones were grown to stationary phase (~10^8^/ml) and diluted to the desired inoculum density. Six-to-eight-week-old female C3H/HeN mice were purchased (Envigo) and six-week-old male NOD-*scid* IL2Rgamma^null^ (NSG) mice were bred at the UCF vivarium from purchased breeding pairs (The Jackson Laboratory). Groups of 6 mice each were needle inoculated with 10^4^ *B*. *burgdorferi* intradermally (C3H/HeN) or with the dose split 4:1 between the intraperitoneal and subcutaneous routes (NSG). Inoculum densities were confirmed by plating for individuals in solid medium or by limiting dilution in liquid medium in 96-well plates. All inoculum cultures were verified to contain the expected endogenous plasmids [76,77] and individuals from each inoculum clone were analyzed for the presence of virulence plasmids lp25, lp28-1 and lp36 [89]. Mouse infection was determined by spirochete reisolation from tissues and quantitative PCR for *B*. *burgdorferi* loads in tissues [35,77].

### Determination of *B*. *burgdorferi* density in blood

On day 6 post-inoculation approximately 50 μl of blood was collected from the submandibular vein of each mouse into K2 EDTA MiniCollect blood collection system tubes (Greiner). For each blood sample, two volumes of blood, approximately 38 μl and 4.8 μl, were each added to 40 ml of liquid BSKII containing RPA cocktail (60 μM rifampicin, 110 μM phosphomycin, and 2.7 μM amphotericin B) and plated for colony forming units (CFU) across two 96-well plates. Plates were incubated at 35°C with 2.5% CO_2_ for 14 days and scored for the presence or absence of spirochete growth. Each positive well was considered a single CFU. *B*. *burgdorferi*/ ml of blood was calculated as the number of CFUs/ volume of blood analyzed multiplied by 100 for each sample and the average of the two measurements for each sample was determined.

### Infection of *Ixodes* larvae by immersion and tick feeding

Approximately 4-month-old *Ixodes scapularis* naïve larval ticks (CDC, BEI resources or Oklahoma State University) were dehydrated by exposure to saturated ammonium sulfate for 24–48 hr. Log phase grown *B*. *burgdorferi* clones were diluted to 2 x 10^7^ cells/ml in BSKII. 500 μl of spirochetes were incubated with dehydrated ticks at 35°C for ~1.5 hrs and washed twice with PBS [92]. The inoculum cultures were verified to contain the expected endogenous plasmids [76,77]. Infectious plasmids lp25, lp28-1 and lp36 were present in 80–100% of individuals of each inoculum culture. Three days post artificial infection cohorts of ~150 larvae were fed to repletion on groups of 3–7 naïve C3H/HeN mice (Envigo) per *B*. *burgdorferi* clone. Subset of fed larvae were allowed to molt into nymphs and subsequently cohorts of 25 nymphs fed on groups of 3–7 naïve C3H/HeN mice (Envigo) per *B*. *burgdorferi* clone. Three weeks post nymph feeding, mice were assayed for infection by spirochete reisolation from tissues [35,77].

### Determination of *B*. *burgdorferi* densities in ticks

The *B*. *burgdorferi* densities in ticks were assessed before and after feeding to repletion on mice by performing qPCR analysis of total DNA isolated from ticks or plating of dilutions of whole triturated ticks in solid or liquid medium [77,89,93]. Total DNA was isolated from groups of 17–50 unfed larvae, 5–13 fed larvae 7–14 days post feeding, and 6–10 unfed nymphs. Ticks were homogenized with a plastic pestle in a microfuge tube, and genomic DNA was isolated using a NucleoSpin tissue kit (Clontech Laboratories) according to the manufacturer’s specifications. Individual fed nymphs were surface sterilized and assessed for colony forming units in solid or liquid medium containing RPA cocktail [89,90].

### Low serum/fatty acid growth analysis

For initial assessment of *B*. *burgdorferi* growth in solid medium, in biological triplicate, *B*. *burgdorferi* clones were inoculated into complete BSKII liquid medium at a starting density of 1x10^5^ spirochetes/ml. The densities of the cultures were determined via Petroff-Hausser count every 24 hours over a period of 96 hours. At each time point an aliquot of each culture was removed and dilutions plated in complete solid medium and by limiting dilution in complete liquid medium in 96-well plates. Percent survival in solid medium of each clone was calculated as the percent survival in solid medium [(actual cfu/expected cfu) x 100] normalized to the percent survival in liquid medium [(actual positive wells/expected positive wells) x 100]. All subsequent solid plating experiments were carried out as described above with the following exceptions, *B*. *burgdorferi* culture aliquots were harvested at the 72-hour time point and the serum level in the solid medium was reduced to 1.8% (low serum solid medium). Liquid growth experiments were performed over a 96-hour time course as described above using liquid medium containing 1.8% serum (low serum liquid medium). Oleic acid (Sigma), linoleic acid (Sigma), palmitic acid (Sigma), cholesterol (Sigma) and glyceryl trioleate (Sigma) were solubilized in 70% ethanol at a concentration of 2000 μg/ml. Each lipid was added to the solid or liquid medium at a final concentration of 20 μg/ml in 0.7% ethanol. 0.7% ethanol served as the vehicle alone control.

### Cloning and purification of recombinant BB0562

The wild-type *bb0562* gene was PCR amplified using Q5 polymerase (New England Biolabs) and primers 2643 + 2644 (S1 Table). The serine 34 and/or serine 58 *bb0562* point mutants were generated using overlapping PCR (S1 Table). The PCR fragments were cloned into pET43.1b in *E*. *coli*, generating 6X*his*, *nus* N-terminal and 6X*his* C-terminal fusion constructs. All fusion constructs were verified by restriction digest and Sanger sequencing. The pET43.1b-*bb0562* plasmids were moved to NiCo21 (DE3) *E*. *coli* (New England Biolabs) for protein induction and purification. The *E*. *coli* clones were grown to late logarithmic phase in lysogeny broth shaking at 37°C, chilled at 4°C for 1 hour and induced with 0.5 mM IPTG at 18°C for 16 hours. Cells were harvested by centrifugation at 4°C, resuspended in 10 ml of His-binding buffer (Cytiva) and disrupted by sonication. Total protein lysates were cleared of insoluble debris by centrifugation at 4°C. The soluble lysate was loaded by gravity flow onto nickel beads and washed according to the manufacturer’s instructions (Cytiva). Protein was eluted from the nickel beads in a stepwise fashion using 100 mM to 500 mM imidazole. The 300 mM imidazole fractions were selected for all downstream applications (S6 Fig). The protein samples were moved into PBS using Zeba spin desalting columns (Thermo Fisher Scientific). The proteins were quantified by BCA protein assay (Pierce) and stored at 4°C.

### Lipase activity assay

Lipase activity was determined using the Lipase detection kit III (fluorometric) containing a proprietary long chain fatty acid triglyceride derivative substrate (Abcam). Two micrograms of each recombinant protein, including Nus alone, were used. The assay was performed in triplicate according to the manufacturer’s instruction (Abcam) over a 300-minute time course. The amount in picomoles (pmol) of fluorescent methylresorufin produced by hydrolysis of the lipase substrate by each recombinant protein at each time point was extrapolated from a standard curve. Lipase activity was calculated as pmol/min/μg.

## Supporting information

S1 Fig Deletion mutants of the *bb0562-bb0564* locus. (A) Schematic representation of the *bb0562*-*bb0564* region of the *B*. *burgdorferi* chromosome (Chr). The target gene(s) was replaced with the *flaBp*-*aadA* antibiotic resistance cassette in wild-type *B*. *burgdorferi* (WT) by allelic exchange. A wild-type copy of the target gene(s) was restored in each mutant clone along with the *flgBp-aacC1* antibiotic cassette by allelic exchange. The infection-active promoter, identified via BbIVET (*Bbive67*, red arrow), and primary transcription start site, identified by 5’RNA-seq (pTSS, light blue arrow), for gene *bb0562* [28] are indicated. Numbers and small arrows indicate approximate locations and orientation of the primers used for clone verification. (B) PCR analysis of the *B*. *burgdorferi* mutant and complement clones. Genomic DNA was isolated from all clones and used as template in PCR reactions, as indicated above the image. A no template control (NTC) served as the negative control. The primer pairs used to amplify the target DNA sequences, given underneath the image, correspond to the labels in panel A. Target DNA sequences are separated by a DNA ladder and fragment sizes, in base pairs (bp), are indicated to the left of the image.
(TIF)

S2 Fig Gene expression analysis of *B. burgdorferi* mutant and complement clones. (A-F) Reverse transcription (RT)-qPCR analysis of *bb0561*, *bb0562*, *bb0563* and *bb0564* expression in wild-type (WT), gene deletion and complement *B*. *burgdorferi* clones. RNA was isolated from *in vitro* grown log phase *B*. *burgdorferi*. Copy numbers for each gene target were normalized to *recA* mRNA copies. Data are presented as the average of biological triplicate samples ± standard deviation. Expression levels across samples for each target gene were compared by one-way ANOVA with Dunnett’s multiple comparisons test to WT, GraphPad Prism 9.0.0. (*p<0.05, **p<0.01, ***p<0.001, ****p<0.0001).
(TIF)

S3 Fig *In vitro* growth analysis of *B. burgdorferi* mutant and complement clones. (A-F) In triplicate *B*. *burgdorferi* clones were grown in complete liquid medium at 35°C. Spirochete density was determined every 24 hours by Petroff-Hausser count under dark field microscopy over a time course of 96–120 hours. Data points represent the average ± standard deviation.
(TIF)

S4 Fig Gene *bb0562* is sufficient to restore wild-type levels of spirochete load in tissues to Δ*bb0562-bb0564 B. burgdorferi*. Groups of 6 immunocompetent C3H/HeN mice were needle inoculated intradermally with 10^4^ *B*. *burgdorferi* per clone. Three weeks post-inoculation ear, heart and joint tissues were collected, and total DNA was extracted. *B*. *burgdorferi* load was measured by quantifying *B*. *burgdorferi flaB* copies normalized to 10^6^ mouse *nid* copies using quantitative PCR. Each data point represents an individual mouse. The mean is indicated by a horizontal black line. Statistical analyses were performed using the unpaired t-test, GraphPad Prism 9.0.0.
(TIF)

S5 Fig Gene *bb0562* is critical for *B. burgdorferi* growth in solid medium. (A) Triplicate cultures of each *B*. *burgdorferi* clone were grown in complete BSKII liquid medium and the densities of the cultures were determined via Petroff-Hausser count under dark field microscopy every 24 hours over a time course of 168 hours. (B-F) At each indicated time point an aliquot of each culture was removed, and dilutions plated in complete solid medium and by limiting dilution in complete liquid medium in 96 well plates. Data represent the average ± standard deviation. Statistical analyses were performed using the one-way ANOVA with Dunnett’s multiple comparisons test to WT, GraphPad Prism 9.0.0 (**p<0.01, ***p<0.001).
(TIF)

S6 Fig Recombinant BB0562 proteins. Purified wild-type (WT) recombinant Nus-BB0562, single (S34A; S34T; S58A; S58T) and double mutant (S34A, S58A; S34T, S58T) proteins and Nus alone, were separated by SDS-PAGE. Proteins were visualized by Coomassie blue staining (CB). Molecular weights are shown in kilodaltons (kDa).
(TIF)

S1 Table Oligonucleotides and probes used in this study. (DOCX)

S2 Table *B. burgdorferi* lacking *bb0562* are attenuated for infection in immunocompetent and immunodeficient mice. (DOCX)

S1 Data Excel spreadsheet containing, in separate sheets, the underlying numerical data and statistical analysis for Figure panels 1C, 2A, 2B, 2C, 2D, 2E, 3A, 3B, 3C, 3D, 3E, 4A, 4B, 4C, 4D, 4E, 5C, A in S2, B in S2, C in S2, D in S2, E in S2, F in S2, A in S3, B in S3, C in S3, D in S3, E in S3, F in S3, S4, A in S5, B in S5, C in S5, D in S5, E in S5, and F in S5. (XLSX)
