# Supplementary figures and images for: BB0562 is a nutritional virulence determinant with lipase activity important for Borrelia burgdorferi infection and survival in fatty acid deficient environments
Source: PLoS Pathog. 2021 Aug 20;17(8):e1009869. doi: 10.1371/journal.ppat.1009869 (PMC8409650; doi:10.1371/journal.ppat.1009869)

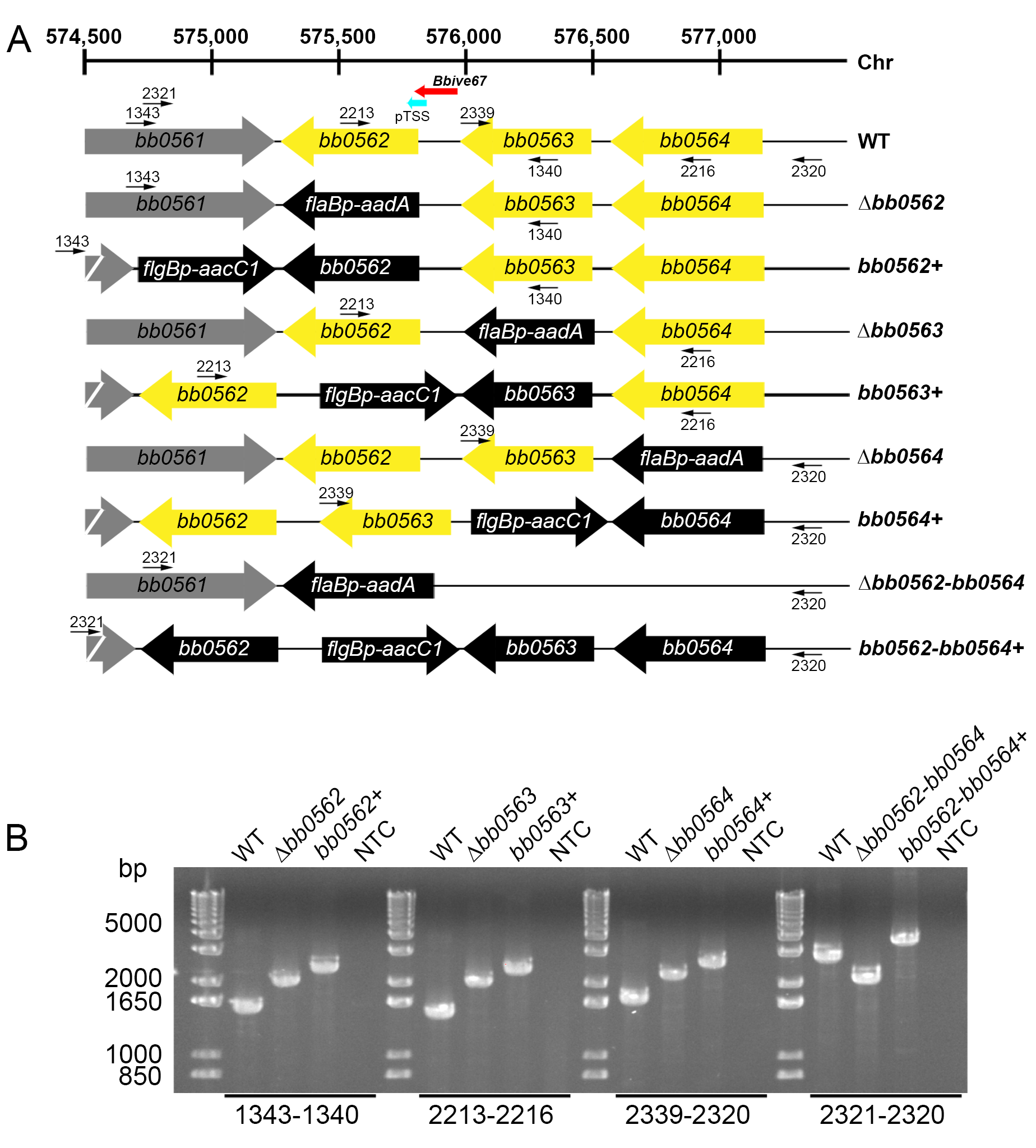

Supplement: S1 Fig — (A) Schematic representation of the bb0562-bb0564 region of the B. burgdorferi chromosome (Chr). The target gene(s) was replaced with the flaBp-aadA antibiotic resistance cassette in wild-type B. burgdorferi (WT) by allelic exchange. A wild-type copy of the target gene(s) was restored in each mutant clone along with the flgBp-aacC1 antibiotic cassette by allelic exchange. The infection-active promoter, identified via BbIVET (Bbive67, red arrow), and primary transcription start site, identified by 5’RNA-seq (pTSS, light blue arrow), for gene bb0562 [28] are indicated. Numbers and small arrows indicate approximate locations and orientation of the primers used for clone verification. (B) PCR analysis of the B. burgdorferi mutant and complement clones. Genomic DNA was isolated from all clones and used as template in PCR reactions, as indicated above the image. A no template control (NTC) served as the negative control. The primer pairs used to amplify the target DNA sequences, given underneath the image, correspond to the labels in panel A. Target DNA sequences are separated by a DNA ladder and fragment sizes, in base pairs (bp), are indicated to the left of the image. (TIF) [file ppat.1009869.s001.tif]

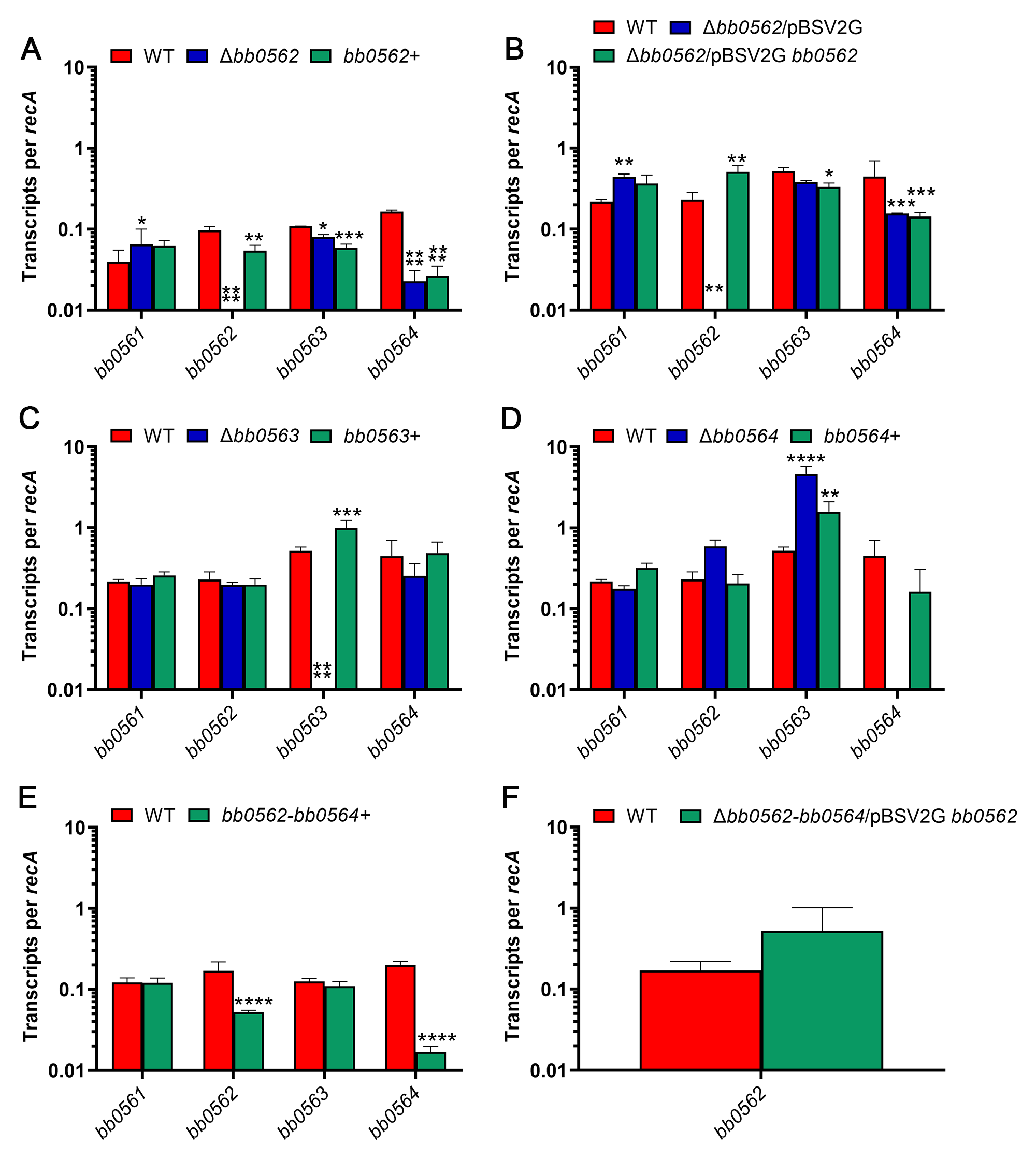

Supplement: S2 Fig — (A-F) Reverse transcription (RT)-qPCR analysis of bb0561, bb0562, bb0563 and bb0564 expression in wild-type (WT), gene deletion and complement B. burgdorferi clones. RNA was isolated from in vitro grown log phase B. burgdorferi. Copy numbers for each gene target were normalized to recA mRNA copies. Data are presented as the average of biological triplicate samples ± standard deviation. Expression levels across samples for each target gene were compared by one-way ANOVA with Dunnett’s multiple comparisons test to WT, GraphPad Prism 9.0.0. (*p<0.05, **p<0.01, ***p<0.001, ****p<0.0001). (TIF) [file ppat.1009869.s002.tif]

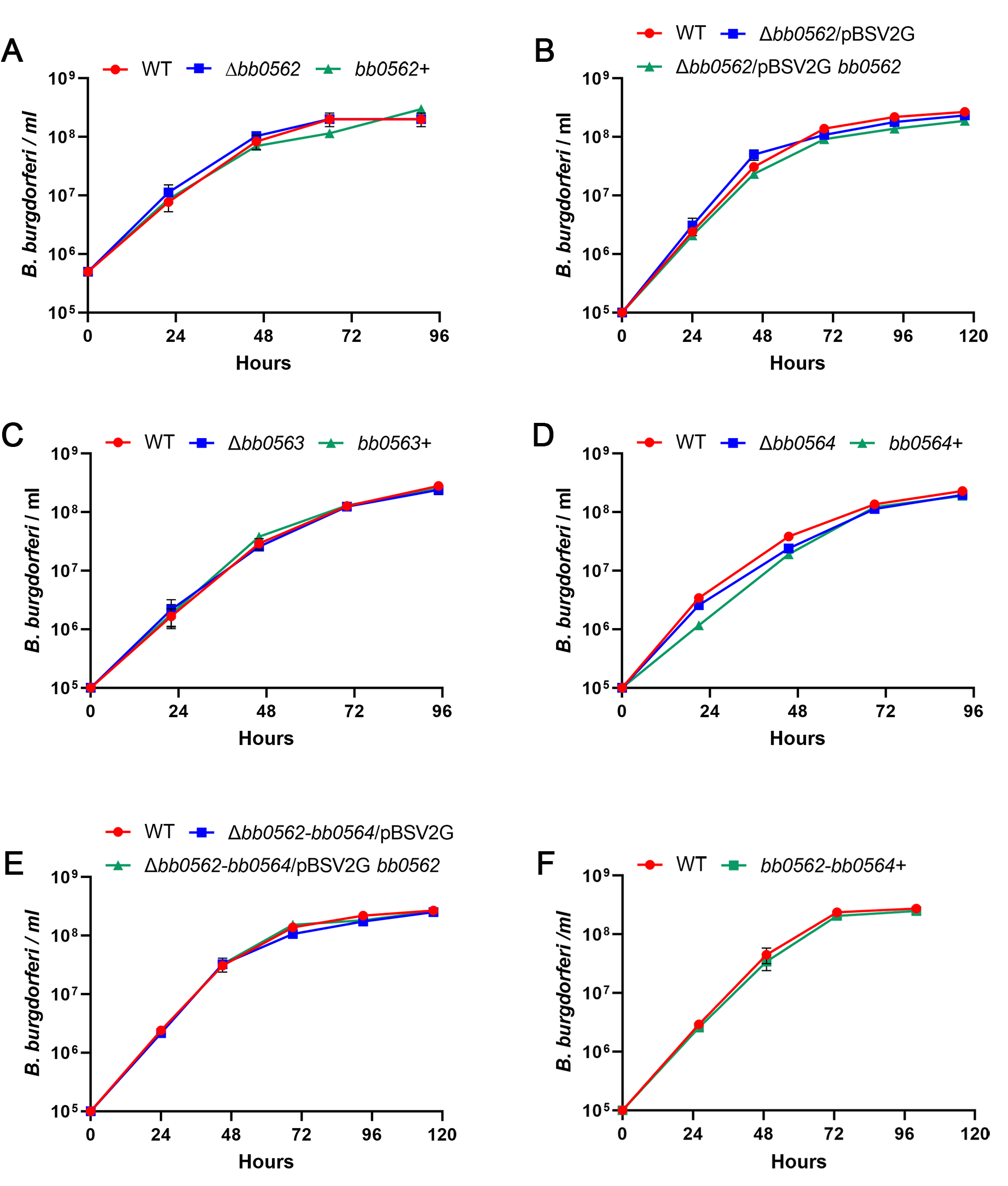

Supplement: S3 Fig — (A-F) In triplicate B. burgdorferi clones were grown in complete liquid medium at 35°C. Spirochete density was determined every 24 hours by Petroff-Hausser count under dark field microscopy over a time course of 96–120 hours. Data points represent the average ± standard deviation. (TIF) [file ppat.1009869.s003.tif]

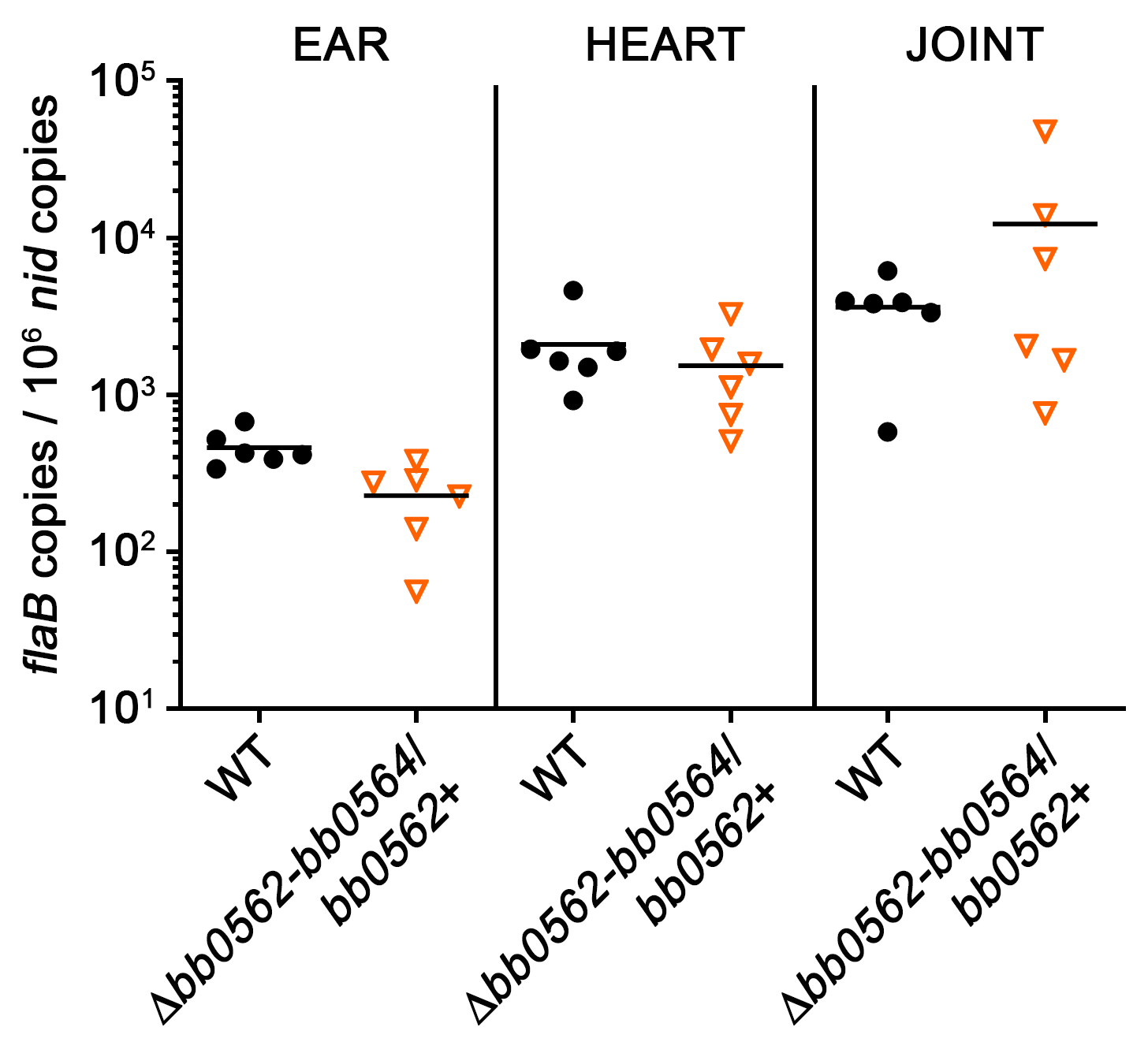

Supplement: S4 Fig — Groups of 6 immunocompetent C3H/HeN mice were needle inoculated intradermally with 104 B. burgdorferi per clone. Three weeks post-inoculation ear, heart and joint tissues were collected, and total DNA was extracted. B. burgdorferi load was measured by quantifying B. burgdorferi flaB copies normalized to 106 mouse nid copies using quantitative PCR. Each data point represents an individual mouse. The mean is indicated by a horizontal black line. Statistical analyses were performed using the unpaired t-test, GraphPad Prism 9.0.0. (TIF) [file ppat.1009869.s004.tif]

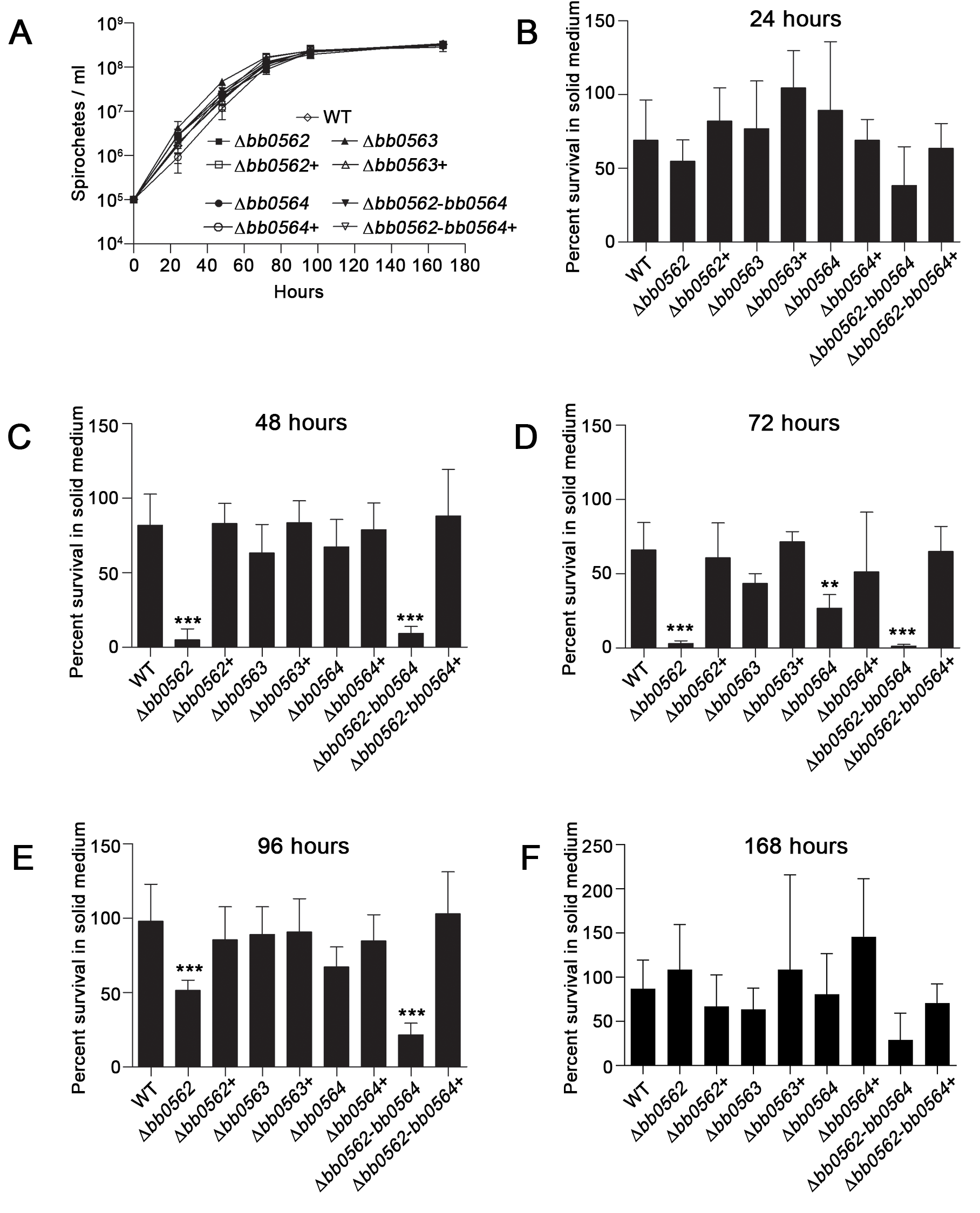

Supplement: S5 Fig — (A) Triplicate cultures of each B. burgdorferi clone were grown in complete BSKII liquid medium and the densities of the cultures were determined via Petroff-Hausser count under dark field microscopy every 24 hours over a time course of 168 hours. (B-F) At each indicated time point an aliquot of each culture was removed, and dilutions plated in complete solid medium and by limiting dilution in complete liquid medium in 96 well plates. Data represent the average ± standard deviation. Statistical analyses were performed using the one-way ANOVA with Dunnett’s multiple comparisons test to WT, GraphPad Prism 9.0.0 (**p<0.01, ***p<0.001). (TIF) [file ppat.1009869.s005.tif]

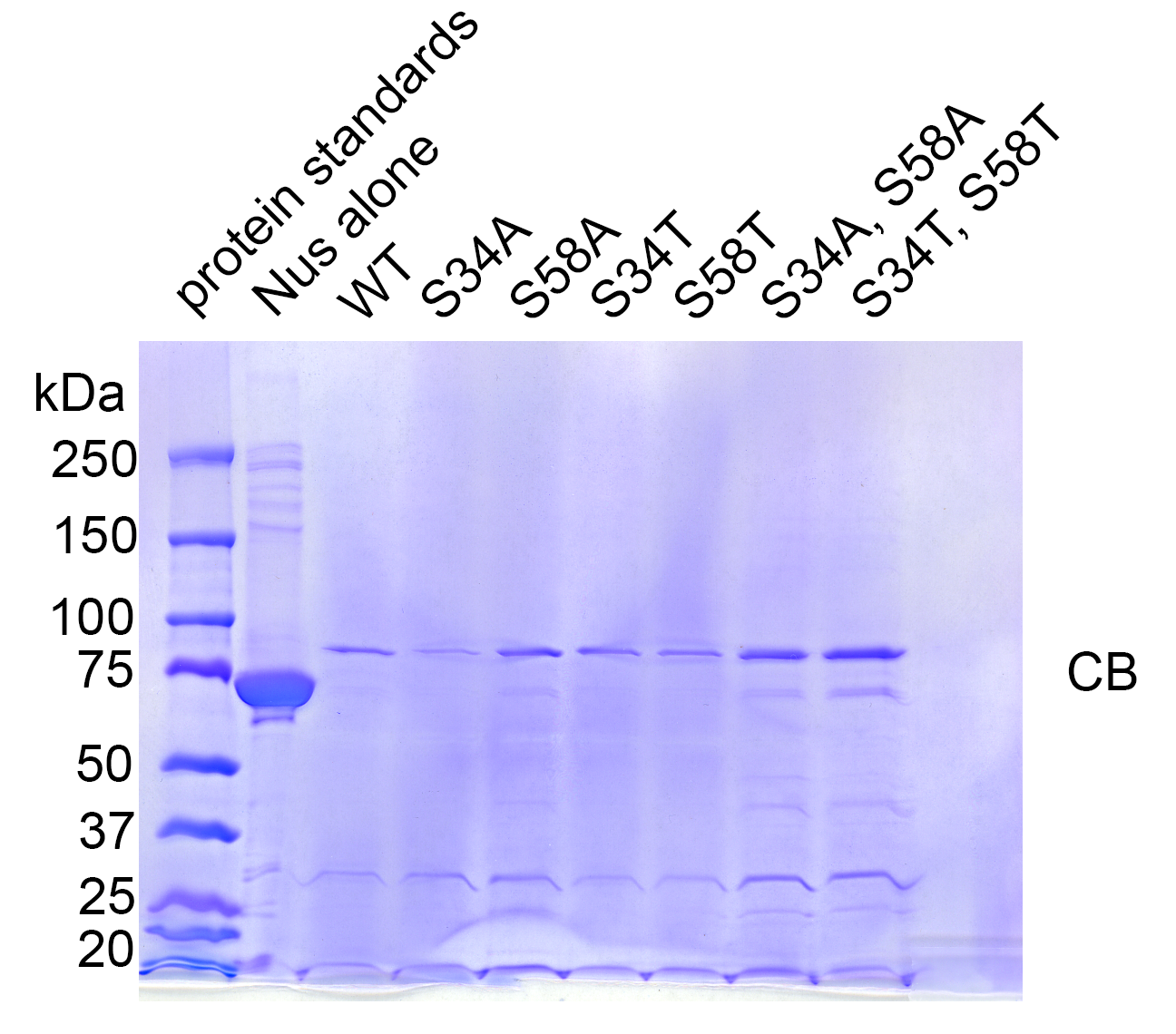

Supplement: S6 Fig — Purified wild-type (WT) recombinant Nus-BB0562, single (S34A; S34T; S58A; S58T) and double mutant (S34A, S58A; S34T, S58T) proteins and Nus alone, were separated by SDS-PAGE. Proteins were visualized by Coomassie blue staining (CB). Molecular weights are shown in kilodaltons (kDa). (TIF) [file ppat.1009869.s006.tif]
