# Supplementary material for: BB0562 is a nutritional virulence determinant with lipase activity important for Borrelia burgdorferi infection and survival in fatty acid deficient environments
Source: PLoS Pathog. 2021 Aug 20;17(8):e1009869. doi: 10.1371/journal.ppat.1009869 (PMC8409650; doi:10.1371/journal.ppat.1009869)
Supplement: S1 Table — (DOCX) [file ppat.1009869.s007.docx]

| **S1 Table.** Oligonucleotides and probes used in this study. | | |  |
| --- | --- | --- | --- |
| Number | Name | Sequence (5’-3’) | Application |
| Primers |  |  |  |
| 1123 | *recA* qPCR 5' | AATAAGGATGAGGATTGGTG | Gene expression |
| 1124 | *recA* qPCR 3' | GAACCTCAAGTCTAAGAGATG | Gene expression |
| 1875 | *flaB* qPCR 5' | GCATTAATCTTACCAGAAACTCC | Gene expression |
| 1876 | *flaB* qPCR 3' | GCATTAACGCTGCTAATCTTAG | Gene expression |
| 2138 | *ospA* qPCR 5' | CGATCTAGGTCAAACCACA | Gene expression |
| 2139 | *ospA* qPCR 3' | GTTCCGTCTGCTCTTGT | Gene expression |
| 2203 | *ospC* qPCR 5' | ACGGATTCTAATGCGGTTTTACCT | Gene expression |
| 2204 | *ospC* qPCR 3' | CAATAGCTTTAGCAGCAATTTCATCT | Gene expression |
| 2374 | *bb0561* qPCR 5’ | GATTATTGCTAGCGATTTACTTAAG | Gene expression |
| 2373 | *bb0561* qPCR 3’ | CAAATCCCAACCATAATTCAC | Gene expression |
| 1715 | *bb0562* qPCR 5' | GGTGGTAGTAATGGAATAAATCTATC | Gene expression |
| 1716 | *bb0562* qPCR 3' | ATATGCTTATATTTCCTCCTATTCC | Gene expression |
| 2179 | *bb0563* qPCR 5' | GGTCTTTACAGCGGAGTAA | Gene expression |
| 2180 | *bb0563* qPCR 3' | CTGCCTCCAAATCTTGAAA | Gene expression |
| 2263 | *bb0564* qPCR 5' | GCTCAGATTTAACAAATGTTTCT | Gene expression |
| 2264 | *bb0564* qPCR 3' | GTTAGGCATTTGGCCA | Gene expression |
| 1650 | *bb0562* 5' +145 nt upstream TSS, KpnI | GGGGTACCATAATAAAATTCTTTTTTAAAAAGTTTTAAAAAAGAAC | *bb0562* *in trans* complement |
| 1649 | *bb0562* 3' Comp SalI | GCGTCGACTTAAAAGATATAGTACTTAGC | *bb0562* *in trans* complement |
| 1764 | *bb0562* 3' + FLAG 3X, SalI | GCGTCGACTTATTTATCATCATCATCTTTATAATCTTTATCATCATCATCTTTATAATCTTTATCATCATCATCTTTATAATCAAAGATATAGTACTTAGCAAATATTCC | BB0562-3XFLAG |
| 1255 | *flaBp-aadA* 5' | TGTCTGTCGCCTCTTGTG | Allelic exchange |
| 1256 | *flapBp-aadA* 3' | TTATTTGCCGACTACCTTGGTG | Allelic exchange |
| 1789 | *flgBp-aacC1* 5' | TAATACCCGAGCTTCAAGGAAGATTTCCTATTAAGGTTGAAC | Allelic exchange |
| 1790 | *flgBp-aacC1* 3' | TTAGGTGGCGGTACTTGGGTCGATATCAAAGTGCATC | Allelic exchange |
| 1340 | *bb0562 +* ~500 bp 5' | CTTGACATTGGTCTTTACAGCG | Allelic exchange Δ*bb0562*, *bb0562* comp |
| 1341 | *bb0562 +* ~500 bp 3' *flaBp* overlap | CGGAAGCCACAAGAGGCGACAGACACGTTGTCTCCCAAATTTTGTC | Allelic exchange Δ*bb0562* |
| 1342 | *bb0562* + ~500 bp 5' *aadA* overlap | GGCGAGATCACCAAGGTAGTCGGCAAATAAGCTGGGGGAATATTTGCTAAG | Allelic exchange Δ*bb0562*, Δ*bb0562-bb0564* |
| 1343 | *bb0562* + ~500 bp 3' | TGAGTTTGAGCTTAGTGATTGG | Allelic exchange Δ*bb0562*, *bb0562* comp |
| 1788 | *bb0562 +* ~500 bp *3' flgB* overlap | GTTCAACCTTAATAGGAAATCTTCCTTGAAGCTCGGGTATTATTATTGCCAAGCGCTATGAAAAAAT | Allelic exchange *bb0562* comp |
| 1791 | *bb0562* 3' aacC1 overlap | GATGCACTTTGATATCGACCCAAGTACCGCCACCTAAATTTAATAAATTAGAATGAATTGGTGTTAAAAGAT | Allelic exchange *bb0562* comp |
| 2213 | *bb0563* up F | GATGTGTGAGACGATATGCTTATA | Allelic exchange Δ*bb0563*, *bb0563* comp |
| 2214 | *bb0563* up R | GGCGAGATCACCAAGGTAGTCGGCAAATAAGGACTAAGATTCTGGTTTACTTAA | Allelic exchange Δ*bb0563* |
| 2215 | *bb0563* down F | CGGAAGCCACAAGAGGCGACAGACATGCTTTATTATCTCCTAAGTATAGCATTC | Allelic exchange Δ*bb0563* |
| 2216 | *bb0563* down R | CTGCAATAGAATTCAGCATTAATAACTT | Allelic exchange Δ*bb0563*, *bb0563* comp |
| 2337 | *bb0563* dwn comp F | GTTCAACCTTAATAGGAAATCTTCCTTGAAGCTCGGGTATTATTAAGTAAACCAGAATCTTAGTC | Allelic exchange *bb0563* comp |
| 2338 | *bb0563* up comp R | GATGCACTTTGATATCGACCCAAGTACCGCCACCTAATAATAAAATTCTTTTTTAAAAAGTTT | Allelic exchange *bb0563* comp |
| 2319 | *bb0564* KO upstream 3’ | CGGAAGCCACAAGAGGCGACAGACAGTTTTTACTTTGCATTGTTAAC | Allelic exchange Δ*bb0564*, Δ*bb0562-bb0564* |
| 2320 | *bb0564* KO upstream 5’ | GACATAATCTACTAAAATTCCAAG | Allelic exchange Δ*bb0564*, *bb0564* comp, Δ*bb0562-bb0564*, *bb0562-bb0564* comp |
| 2339 | *bb0564* KO downstream 5’ | ATTAAGTAAACCAGAATCTTAG | Allelic exchange Δ*bb0564*, *bb0564* comp |
| 2282 | *bb0564* KO downstream 3’ | GGCGAGATCACCAAGGTAGTCGGCAAATAATTTTCACTTTTTAGATAAAAAAAATTATTG | Allelic exchange Δ*bb0564* |
| 2321 | *bb0562-bb0564* KO downstream 3’ | GATTGGATTTTAGATCTTTATTATG | Allelic exchange Δ*bb0562-bb0564*,  *bb0562-bb0564* comp |
| 2343 | *bb0564* comp down 5’ | GATGCACTTTGATATCGACCCAAGTACCGCCACCTAATTTTCACTTTTTAGATAAAAAAAAT | Allelic exchange *bb0564* comp, *bb0562-bb0564* comp |
| 2344 | *bb0564* comp up 3’ | GTTCAACCTTAATAGGAAATCTTCCTTGAAGCTCGGGTATTACTATATCTTAAGAGAGCCTT | Allelic exchange *bb0564* comp, *bb0562-bb0564* comp |
| 1137 | *flaB*-TaqMan-FWD | TCTTTTCTCTGGTGAGGGAGCT | *B. burgdorferi* quantitation |
| 1138 | *flaB*-TaqMan-REV | TCCTTCCTGTTGAACACCCTCT | *B. burgdorferi* quantitation |
| 1140 | *nid*-TaqMan-FWD | CACCCAGCTTCGGCTCAGTA | *B. burgdorferi* quantitation |
| 1141 | *nid*-TaqMan-REV | TCCCCAGGCCATCGGT | *B. burgdorferi* quantitation |
| 2643 | BB0562 BamHI pET43.1b FWD | CTCGTGGATCCGATGAAAAAAATTTTTATATTGTTTATCATGATTG | Recombinant BB0562 |
| 2644 | BB0562 XhoI pET43.1b REV | GGTGCTCGAGAAAGATATAGTACTTAGCAAATATTCCC | Recombinant BB0562 |
| 2657 | BB0562 S34toA 3' | GTTAATAATTGGATTTCCAAT**TGC**TGCTCC | Recombinant BB0562 |
| 2658 | BB0562 S34toA 5' | GAATTGGCTTTGGAGCA**GCA**ATTGG | Recombinant BB0562 |
| 2659 | BB0562 S34toT 3' | GTTAATAATTGGATTTCCAAT**TGT**TGCTCC | Recombinant BB0562 |
| 2660 | B0562 S34toT 5' | GAATTGGCTTTGGAGCA**ACA**ATTGG | Recombinant BB0562 |
| 2661 | BB0562 S58toA 3' | CTGATAGATTTATTCCATT**TGC**ACCACC | Recombinant BB0562 |
| 2662 | BB0562 S58toA 5' | GAAATTGGCTATGGTGGT**GCA**AATGG | Recombinant BB0562 |
| 2663 | BB0562 S58toT 3' | CTGATAGATTTATTCCATT**TGT**ACCACC | Recombinant BB0562 |
| 2664 | BB0562 S58toT 5' | GAAATTGGCTATGGTGGT**ACA**AATGG | Recombinant BB0562 |
|  |  |  |  |
| Probes |  |  |  |
| 1139 | *flaB*-TaqMan-Probe | 6-FAM-AAACTGCTCAGGCTGCACCGGTTC-TAMRA^a^ | *B. burgdorferi* quantitation |
| 1142 | *nid*-TaqMan-Probe | 6-FAMCGCCTTTCCTGGCTGACTTGGACA-TAMRA^a^ | *B. burgdorferi* quantitation |
| 64 | BB0562 Coding Region | CTACCACCATAGCCAATTTCAAAATCAATGAAAGGAAATGAC | Northern blot |
| PA059 | BB0563 5' Coding Region | CCTGCTCCAAATTTACCCCTTGCAGATTCTTTTGAATTAGAA | Northern blot |
| PA060 | BB0564 Coding Region | CCGAGACACTCAATATTGTTACCCCTACTCCAATATCAAAGT | Northern blot |

^a^6-FAM, 6-carboxyfluorescein; TAMARA, 6-carboxytetramethylrhodamine
