# Supplementary material for: BB0562 is a nutritional virulence determinant with lipase activity important for Borrelia burgdorferi infection and survival in fatty acid deficient environments
Source: PLoS Pathog. 2021 Aug 20;17(8):e1009869. doi: 10.1371/journal.ppat.1009869 (PMC8409650; doi:10.1371/journal.ppat.1009869)
Supplement: S2 Table — (DOCX) [file ppat.1009869.s008.docx]

**S2 Table.** *B. burgdorferi* lacking *bb0562* are attenuated for infection in immunocompetent and immunodeficient mice.

| Mouse Strain | Inoculum | *B. burgdorferi* clone | Spirochete reisolation from tissues^a^ | | | | | |  |
| --- | --- | --- | --- | --- | --- | --- | --- | --- | --- |
|  |  |  | Inoculation  site | Ear | Heart | Bladder | Joint | Total | Percent positive tissues |
| C3H/HeN | 10^4^ intradermal | WT | 22/24 | 21/24 | 24/24 | 24/24 | 24/24 | 117/120 | 98% |
|  |  | Δ*bb0562* | 12/24 | 18/36 | 17/36 | 19/36 | 19/36 | 85/170 | 50% |
|  |  | *bb0562*+ | 18/18 | 18/18 | 18/18 | 18/18 | 18/18 | 90/90 | 100% |
|  |  | Δ*bb0563* | 24/24 | 24/24 | 24/24 | 24/24 | 24/24 | 120/120 | 100% |
|  |  | *bb0563+* | 17/18 | 15/18 | 18/18 | 18/18 | 18/18 | 86/90 | 96% |
|  |  | Δ*bb0564* | 23/24 | 23/24 | 24/24 | 24/24 | 24/24 | 118/120 | 98% |
|  |  | *bb0564*+ | 18/18 | 14/18 | 18/18 | 18/18 | 18/18 | 86/90 | 96% |
|  |  | Δ*bb0562*-*bb0564* | 8/24 | 6/24 | 5/24 | 5/24 | 6/24 | 30/120 | 25% |
|  |  | *bb0562*-*bb0564*+ | 10/12 | 12/12 | 12/12 | 12/12 | 12/12 | 58/60 | 97% |
| C3H/HeN | 10^4^ intradermal | WT | 8/12 | 8/12 | 8/12 | 8/12 | 8/12 | 40/60 | 67% |
|  |  | Δ*bb0562*/v | 2/12 | 8/12 | 0/12 | 3/12 | 5/12 | 18/60 | 30% |
|  |  | Δ*bb0562*/*bb0562*+ | 10/12 | 12/12 | 12/12 | 12/12 | 12/12 | 58/60 | 97% |
|  |  | Δ*bb0562*-*bb0564*/v | 0/12 | 0/12 | 0/12 | 0/12 | 0/12 | 0/60 | 0% |
|  |  | Δ*bb0562*-*bb0564*/*bb0562*+ | 12/12 | 12/12 | 12/12 | 12/12 | 12/12 | 60/60 | 100% |
| NSG | 10^4^ intraperitoneal/ subcutaneous | Δ*bb0562* | n/a^b^ | 2/6 | n/a | 2/6 | 2/6 | 6/18 | 33% |
|  |  | *bb0562^+^* | n/a | 6/6 | n/a | 6/6 | 6/6 | 18/18 | 100% |
| C3H/HeN | Nymph feeding | WT | n/a | 3/5 | 4/4^c^ | 5/5 | 5/5 | 17/19 | 89% |
|  |  | Δ*bb0562* | n/a | 2/7 | 2/7 | 2/7 | 2/7 | 8/28 | 29% |
|  |  | *bb0562^+^* | n/a | 4/4 | 4/4 | 4/4 | 4/4 | 16/16 | 100% |
|  |  | Δ*bb0563* | n/a | 3/3 | 3/3 | 3/3 | 3/3 | 12/12 | 100% |
|  |  | Δ*bb0564* | n/a | 3/3 | 3/3 | 3/3 | 3/3 | 12/12 | 100% |
|  |  | Δ*bb0562-bb0564* | n/a | 1/3 | 1/3 | 1/3 | 1/3 | 4/12 | 33% |

^a^Three weeks post-inoculation mouse tissues were cultured in BSKII medium for 7-10 days and examined under dark-field microscopy for the presence of spirochetes. Number of positive tissues/number of tissues analyzed. Combined data from 1-6 replicate experiments using 3-6 mice per group.

^b^Not applicable, tissue sites were not analyzed.

^c^The reisolation culture of the heart tissue from one of the five mice was not able to be evaluated for spirochete reisolation due to contamination.
